# LPCAT1 reprogramming cholesterol metabolism promotes the progression of esophageal squamous cell carcinoma

**DOI:** 10.1038/s41419-021-04132-6

**Published:** 2021-09-13

**Authors:** Mingyue Tao, Jing Luo, Tong Gu, Xiaojuan Yu, Zhen Song, Yali Jun, Hao Gu, Kairong Han, Xiujuan Huang, Weiyong Yu, Su’an Sun, Zhengwei Zhang, Lu Liu, Xiaofei Chen, Li Zhang, Chao Luo, Qilong Wang

**Affiliations:** 1 Department of Central Laboratory, The Affiliated Huaian No.1 People’s Hospital, Nanjing Medical University, 223300 Huai’an, China; 2 Department of Clinical Oncology, The Affiliated Huaian No.1 People’s Hospital, Nanjing Medical University, 223300 Huaian, P.R. China; 3 Institute of Reproductive Health, Center for Reproductive Medicine, Tongji Medical College, Huazhong University of Science and Technology, 430000 Wuhan, China; 4 Molecular Bioinformatics Group, Faculty of Computer Science and Mathematics, Institute of Computer Science, Frankfurt am Main, Germany; 5 Department of Pathology, The Affiliated Huaian No.1 People’s Hospital, Nanjing Medical University, 223300 Huai’an, China; 6 Biological Sample Bank of Esophageal Cancer, The Affiliated Huaian No.1 People’s Hospital, Nanjing Medical University, 223300 Huai’an, China

**Keywords:** Cancer metabolism, Cancer prevention

## Abstract

Tumor cells require high levels of cholesterol for membrane biogenesis for rapid proliferation during development. Beyond the acquired cholesterol from low-density lipoprotein (LDL) taken up from circulation, tumor cells can also biosynthesize cholesterol. The molecular mechanism underlying cholesterol anabolism in esophageal squamous cell carcinoma (ESCC) and its effect on patient prognosis are unclear. Dysregulation of lipid metabolism is common in cancer. Lysophosphatidylcholine acyltransferase 1 (LPCAT1) has been implicated in various cancer types; however, its role in esophageal squamous cell carcinoma (ESCC) remains unclear. In this study, we identified that LPCAT1 is highly expressed in ESCC and that LPCAT1 reprograms cholesterol metabolism in ESCC. LPCAT1 expression was negatively correlated with patient prognosis. Cholesterol synthesis in ESCC cells was significantly inhibited following LPCAT1 knockdown; cell proliferation, invasion, and migration were significantly reduced, along with the growth of xenograft subcutaneous tumors. LPCAT1 could regulate the expression of the cholesterol synthesis enzyme, SQLE, by promoting the activation of PI3K, thereby regulating the entry of SP1/SREBPF2 into the nucleus. LPCAT1 also activates EGFR leading to the downregulation of INSIG-1 expression, facilitating the entry of SREBP-1 into the nucleus to promote cholesterol synthesis. Taken together, LPCAT1 reprograms tumor cell cholesterol metabolism in ESCC and can be used as a potential treatment target against ESCC.

## Introduction

Esophageal squamous cell carcinoma (ESCC) is the most common type of esophageal cancer, accounting for 90% of all cases [1, 2]. The overall 5-year survival rate of ESCC is <13% after initial diagnosis due to the high rates of recurrence, invasion, and metastasis [3]. Although multiple pathways have reported to be altered in ESCC, including PIK3CA, TP53, KRAS, and epidermal growth factor receptor (EGFR) [4, 5], other molecular mechanisms involved in the initiation, progression, and metastasis of ESCC remain elusive [6–8]. As early detection is critical for improving ESCC patient outcome and reducing disease mortality, there is an urgent need to identify novel targets and effective markers for ESCC.

Previous studies have described the activation of lipid biosynthesis and lipid remodeling in cancer cells [9]. The phospholipid biosynthesis/remodeling enzyme lysophosphatidylcholine acyltransferase 1 (LPCAT1) is a key enzyme in the lipid remodeling pathway known as the Lands’ cycle [10–12]. LPCAT1 is implicated in various cancer types; overexpression of *LPCAT1* was recently described in colorectal cancer, prostate cancer, lung cancer, and clear cell renal cell carcinomas [13–16]. In addition, overexpression of *LPCAT1* also led to a significant growth advantage in cultured colorectal cancer cells [13]. Persistent survival signaling is a hallmark of cancer [17]; amplified and mutated growth factor receptors and associated mutated signaling proteins reside primarily within or at the plasma membrane and enable tumor cells to become independent of external growth control cues [18]. The plasma membrane of cancer cells is hypothesized to significantly change its structure and organization to support factor signaling because the composition of phospholipids in the membrane can impact intracellular signaling by controlling membrane architecture [19]. The signaling activity of the plasma membrane is influenced by its biophysical properties, including curvature, charge, fluidity, and local architecture [20]. These biophysical properties depend on specific constituents of the lipid bilayer, including phospholipids and cholesterol [21, 22]. However, the molecular mechanisms that aid the establishment of plasma membrane composition and structure in esophageal cancer are not well understood.

Growth factor receptor signaling alters nutrient uptake and metabolic pathway flux, which can determine the specific lipid composition of the cellular membranes and regulate their shape and fluidity, as well as the corresponding clustering and activation of the signaling complexes [23]. Previous work has demonstrated that altered growth factor receptor signaling in cancer can determine the levels of major lipid classes and their precursors [24], yet the specific enzymes responsible for mediating these lipid changes and how they enhance esophageal cancer remain poorly understood.

Here we used molecular and bioinformatic approaches to investigate the critical enzymatic factors of the growth factor signaling system and components of plasma membrane reorganization in esophageal cancer. We further characterized the role of the lipid metabolic enzyme LPCAT1 in ESCC tumor growth and poor patient outcome and posit that LPCAT1 is a potential target for esophageal cancer diagnosis and treatment.

## Results

### *LPCAT1* is overexpressed in ESCC and correlated with poor prognosis in ESCC patients

**Fig. 1:**
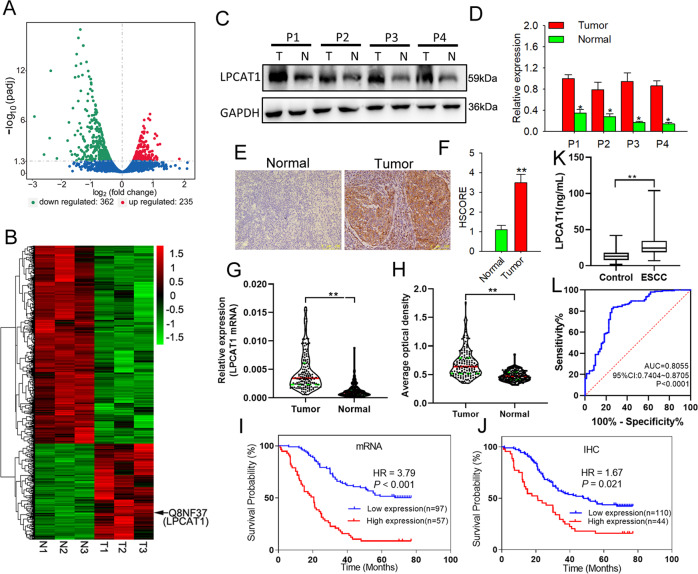
LPCAT1 is highly upregulated in esophageal squamous cell carcinoma and correlates with poor patient prognosis. **A** Volcano plot compared the expression fold changes of the genes for ESCC tissues versus adjacent normal tissues. The red dots represent the genes with significantly changed expression level. **B** Clustered heat map for all genes with altered expression, with rows representing gene and columns representing tissues. **C**, **D** Expression levels of LPCAT1 in ESCC tumor and normal tissues were determined using western blotting analysis. Data are shown as relative expression means and *P* value from three independent experiments. **E** Expression levels of LPCAT1 in tumors were determined using immunohistochemistry analysis. Histograms show the means and *P* value from three independent experiments. **F** Statistical analyses of the LPCAT1 in tumor tissues. **G** The mRNA of LPCAT1 in 185 ESCC and normal tissues was determined using qRT-PCR, **P < 0.01 via unpaired *t* test. **H** The protein of LPCAT1 in 185 ESCC and normal tissues was determined using immunohistochemistry analysis, **P < 0.01 via unpaired *t* test. **I** Statistical analyses of the association between LPCAT1 expression and survival in patients with ESCC. **J** Statistical analyses of the association of LPCAT1 expression with survival probability in ESCC patients. **K** Serum LPCAT1 levels in 71 healthy subjects and 154 patients with ESCC. *P* < 0.01 via unpaired *t* test. **L** ROC curve analysis of the value of LPCAT1 in ESCC diagnosis. *P < 0.05. **P < 0.01.

To investigate *LPCAT1* expression in ESCC, we compared *LPCAT1* expression profile in ESCC tissues and their matched adjacent normal tissues using mass spectrographic analysis. We found that 235 genes were upregulated and 362 genes were downregulated in ESCC tissues relative to the normal tissue (Fig. 1A). The gene ontology (GO) and Kyoto Encyclopedia of Genes and Genomes pathway enrichment analyses of these genes were also assessed, which showed that the functions of proteolysis, tight junction, focal adhesion, and invasion of epithelial cells were enriched in ESCC cells relative to normal tissues (Supplementary Fig. 1A, B). We also performed hierarchical clustering of the upregulated and downregulated genes, which showed that the expression of *LPCAT1* was much higher in ESCC tissues than in normal tissues (Fig. 1B). Among the differentially expressed genes, *LPCAT1* was highly upregulated in ESCC tissues compared to that in normal tissues. To verify this finding, we analyzed the expression of *LPCAT1* in 24 common tumors using the Gene Expression Profiling Interactive Analysis public database analysis platform. These results showed that *LPCAT1* expression in esophageal cancer was upregulated in comparison to the non-cancerous samples (Supplementary Fig. 1C, D), further suggesting that *LPCAT1* is overexpressed in ESCC.

To investigate the clinical significance of *LPCAT1* in ESCC, we collected four pairs of ESCC tissues and their adjacent normal tissues. The western blotting and immunohistochemical (IHC) staining results showed that LPCAT1 was overexpressed in ESCC compared to that in the adjacent normal tissues (Fig. 1C–F). To further confirm this result, we followed up 154 primary ESCC patients for 5 years (Supplementary Fig. 1E). Clinical information for the patients with ESCC is summarized in Table 1. The results showed that the gene expression (Fig. 1G) and protein levels (Fig. 1H) of LPCAT1 were significantly higher in tumor tissues than in normal tissues adjacent to cancer in patients with ESCC. In addition, we found that the expression of *LPCAT1* was correlated with the prognosis of lymph node staging of ESCC patients (Table 2 and Supplementary Fig. 1F, G). Consistent with this result, when patients were divided into two groups based on *LPCAT1* expression, the patients with higher levels of *LPCAT1* had a significantly shorter rate of survival (Fig. 1I, J). In addition, the serum levels of LPCAT1 in normal healthy individuals and 154 patients with ESCC showed that LPCAT1 is high in patients with ESCC and may be a potential diagnostic tool for ECSS (Fig. 1K, L). These data confirmed that the overexpression of LPCAT1 is correlated with poor patient prognosis.

**Table 1:** Characteristics of subjects in screening and validation stages.

Characteristics	Gene expression sample				
	Dead	Alive	OS (%)	HR (95% CI)^a^	*P* value^a^
	(*N* = 100)	(*N* = 54)			
Gender					0.995
Male	66 (66.00%)	41 (75.93%)	38.32%	1.00	
Female	34 (34.00%)	13 (24.07%)	27.66%	1.00 (0.65, 1.55)	
Age					
≤60 years	32 (32.00%)	17 (31.48%)	34.69%	1.00	0.358
>60 years	68 (68.00%)	37 (68.52%)	35.24%	1.23 (0.79, 1.92)	
T stage					0.046
T1–2	23 (23.00%)	22 (40.74%)	48.89%	1.00	
T3–4	77 (77.00%)	32 (59.26%)	29.36%	1.68 (1.01, 2.80)	
N stage					
N_0_	46 (46.00%)	44 (81.48%)	48.89%	1.00	<0.001
N_+_	54 (54.00%)	10 (18.52%)	15.63%	2.44 (1.60, 3.70)	
Differentiation					
G1	32 (32.00%)	25 (46.3%)	43.86%	1.00	
G2	61 (61.00%)	26 (48.15%)	26.89%	1.20 (0.74, 1.83)	0.429
G3	7 (7.00%)	3 (5.56%)	30%	1.33 (0.57, 311)	0.514
Location					
Upper	5 (5.00%)	5 (9.26%)	50%	1.00	
Middle	73 (73.00%)	28 (51.85%)	27.72%	1.97 (0.78, 4.99)	0.152
Lower	22 (22.00%)	21 (38.89%)	48.84%	0.78 (0.28, 2.17)	0.64
LPCAT1 (qPCR)					<0.001
Low expression	48 (48.00%)	49 (90.74%)	50.52%	1.00	
High expression	52 (52.00%)	5 (9.26%)	8.77%	3.79 (2.46, 5.84)	
LPCAT1 (IHC)					0.021
Low expression	63 (63%)	38 (87.04%)	37.62%	1.00	
High expression	37 (37%)	16 (12.96%)	30.19%	1.67 (1.08, 2.59)	

^a^Based on Cox proportional hazards regression analysis, gender, age, TNM stage, tumor differentiation, and tumor location were adjusted when appropriate.

**Table 2:** Association between LPCAT1 expression (based on mRNA expression and protein expression) and clinicopathological factors.

Factors	*N* = 154	mRNA expression			Protein expression		
		Negative (*n* = 97)	Positive (*n* = 57)	*P* value^a^	Negative (*n* = 110)	Positive (*n* = 44)	*P* value^a^
Gender				0.561			0.543
Male	107	69 (71.13%)	38 (66.67%)		78 (68.83%)	29 (65.91%)	
Female	47	28 (28.87%)	19 (33.33%)		32 (31.17%)	15 (34.09%)	
Age				0.961			0.702
≤60 years	49	31 (31.96%)	18 (31.58%)		36 (32.73%)	13 (29.55%)	
>60 years	105	66 (68.04%)	39 (68.42%)		74 (67.27%)	31 (70.45%)	
T stage				0.180			0.265
T1–2	45	32 (32.99%)	13 (22.81%)		35 (31.82%)	10 (22.73%)	
T3–4	109	65 (67.01%)	44 (77.19%)		75 (68.18%)	34 (77.27%)	
N stage				0.033			0.039
N_0_	90	63 (64.95%)	27 (47.37%)		70 (63.64%)	20 (45.45%)	
N_+_	64	34 (35.05%)	30 (52.63%)		40 (36.36%)	24 (54.55%)	
Differentiation				0.376			0.920
G1	57	40 (41.24%)	17 (29.82%)		42 (38.18%)	15 (34.09%)	
G2	87	51 (52.58%)	36 (63.16%)		61 (55.45%)	26 (59.09%)	
G3	10	6 (6.19%)	4 (7.02%)		7 (6.36%)	3 (6.82%)	
Location				0.326			0.321
Upper	10	4 (4.12%)	6 (10.53%)		9 (8.18%)	1 (2.27%)	
Middle	101	65 (67.07%)	36 (63.16%)		73 (66.36%)	28 (63.64%)	
Lower	43	28 (28.87%)	15 (26.32%)		28 (25.45%)	15 (34.09%)	

^a^Based on *χ*^2^ test or Fisher’s exact test if necessary.

### LPCAT1 promotes ESCC cell proliferation, migration, and invasion

**Fig. 2:**
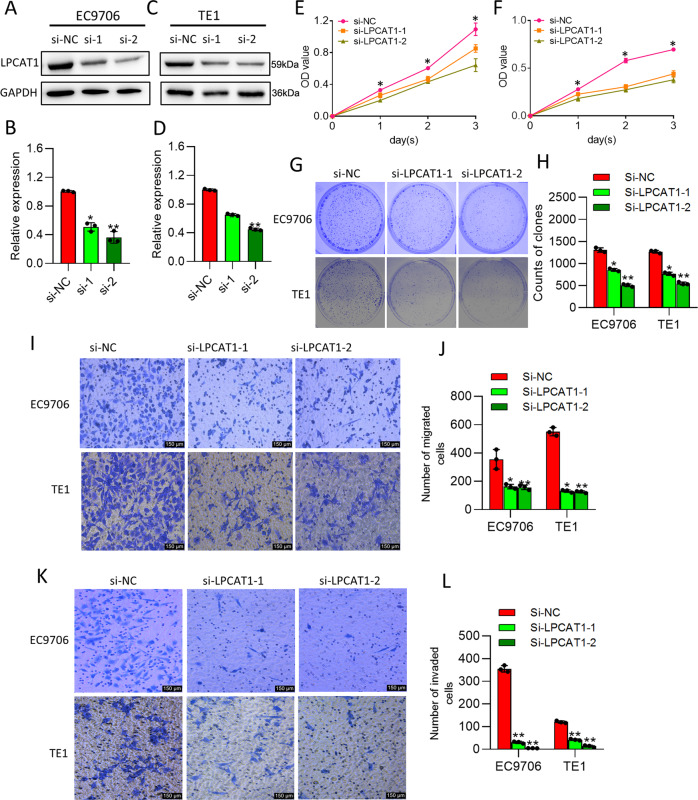
LPCAT1 promotes ESCC cell proliferation, migration, and invasion. **A**–**D** PCR and western blotting showing the expression of LPCAT1 in EC9706 cells (**A**, **B**) and TE1 cells (**C**, **D**) transfected with si-NC and si-LPCAT1. Data are shown as relative expression means and *P* value from three independent experiments. **E**, **F** The proliferative ability of EC9706 cells (**E**) and TE1 cells (**F**) after transfection was evaluated using CCK-8 assay. **G**, **H** Colony-formation assay for LPCAT1-knockdown EC9706 cells and TE1 cells (**G**) and quantitative analysis of LPCAT1 in each group (**H**). **I**, **J** Transwell chamber assays for LPCAT1-depletion ESCC cells. The average numbers of migrated cells were counted after 24 h incubation and expressed as mean ± S.D. **K**, **L** Matrigel invasion assays for LPCAT1-depletion ESCC cells. The average numbers of migrated cells were counted after 24 h incubation and expressed as mean ± S.D. Data are from three independent experiments. *P < 0.05, **P < 0.01 (one-way ANOVA).

We have shown that expression of LPCAT1 at both the gene and protein levels is much higher in ESCC cells than that in the normal esophageal epithelial cells (Supplementary Fig. 2A–C). To evaluate the roles of LPCAT1 in ESCC, we first carried out depletion of LPCAT1 with small interfering RNA (siRNA) that resulted in a significant knockdown of *LPCAT1* in EC9706 cells (Fig. 2A, B) and TE1 cells (Fig. 2C, D). The proliferation of ESCC cells was decreased after LPCAT1 knockdown (Fig. 2E, F). We further investigated whether LPCAT1 contributes to tumor progression in esophageal cancer; the clonogenic ability of EC9706 and TE1 cells with or without the LPCAT1 knockdown showed that LPCAT1 promoted ESCC cell proliferation (Fig. 2G, H). However, whether LPCAT1 can affect cell migration and invasion was unclear; therefore, we next investigated the migration of EC9706 and TE1 cells transfected with or without LPCAT1 knockdown using the Transwell (Fig. 2I, J) and wound healing assays (Supplementary Fig. 3), as well as their invasion using the Transwell assay (Fig. 2K, L). The results consistently demonstrated that the ESCC cells overexpressing *LPCAT1* exhibited enhanced proliferation, migration, and invasion abilities compared to cells that did not overexpress *LPCAT1*.

### LPCAT1 inhibits apoptosis of ESCC cells and promotes cell cycle progression and anoikis resistance

**Fig. 3:**
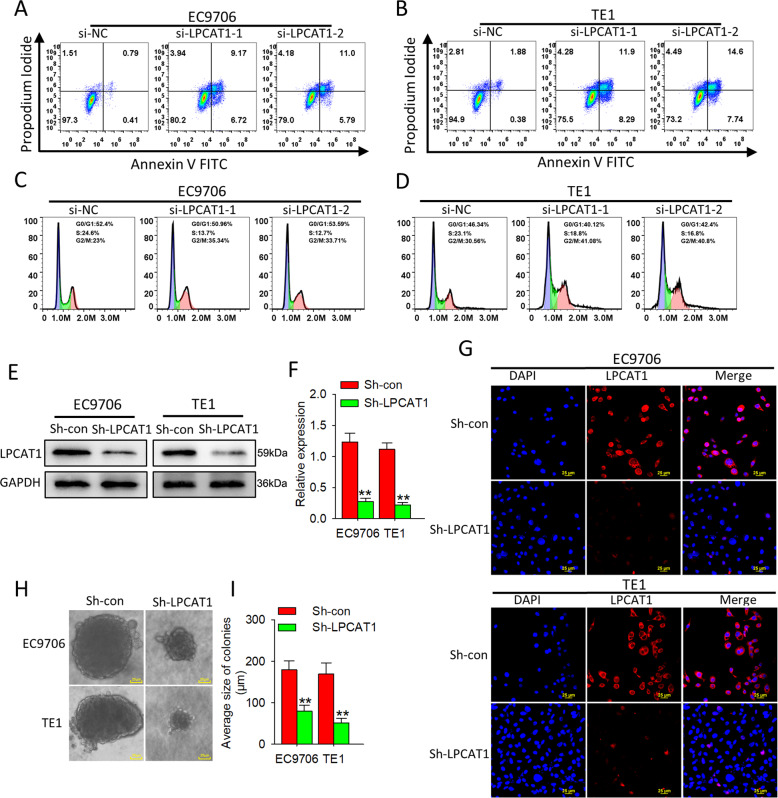
LPCAT1 inhibits apoptosis of ESCC cells and promotes cell cycle progression and anoikis resistance. **A**, **B** Annexin V-FITC/PI double staining was used to assess the apoptosis of EC9706 cells and TE1 cells after transfection with si-NC and si-LPCAT1. **C**, **D** DNA content of EC9706 cells and TE1 was analyzed by flow cytometry after transfection with si-NC and si-LPCAT1. **E**, **F** EC9706 cells and TE1 cells were stably transfected with LPCAT1 lentivirus. **G** Confocal assay was performed to assess the expression of LPCAT1 in EC9706 cells and TE1 cells. **H**, **I** Colony-formation assay was performed to assess EC9706 cells and TE1 cells for anoikis resistance. The average size of colonies was calculated as mean ± S.D. **P < 0.01 (unpaired *t* test). Data are from three independent experiments.

To further investigate whether LPCAT1 inhibits apoptosis in ESCC cells, Annexin V and propidium iodide (PI) were detected in ESCC cells that did or did not overexpress *LPCAT1* using flow cytometry (Fig. 3A, B). Flow cytometry-based investigation of cell cycle distribution after *LPCAT1* knockdown in two ESCC cell lines showed more cells in G2/M phase and less cells in S phase (Fig. 3C, D). Anoikis resistance is the first step toward tumor metastases but whether LPCAT1 affects anoikis in ESCC cells was unknown. We transfected LPCAT1 lentivirus into ESCC cells and detected the expression of LPCAT1 by western blot (Fig. 3E, F) and confocal microscopy (Fig. 3G) and found that LPCAT1 promoted anoikis resistance in ESCC cells (Fig. 3H, I). Taken together, these results suggest that LPCAT1 inhibits apoptosis and promotes cell cycle progression and anoikis resistance in ESCC cells.

### LPCAT1 promotes ESCC progression through the cholesterol metabolism signaling pathway

**Fig. 4:**
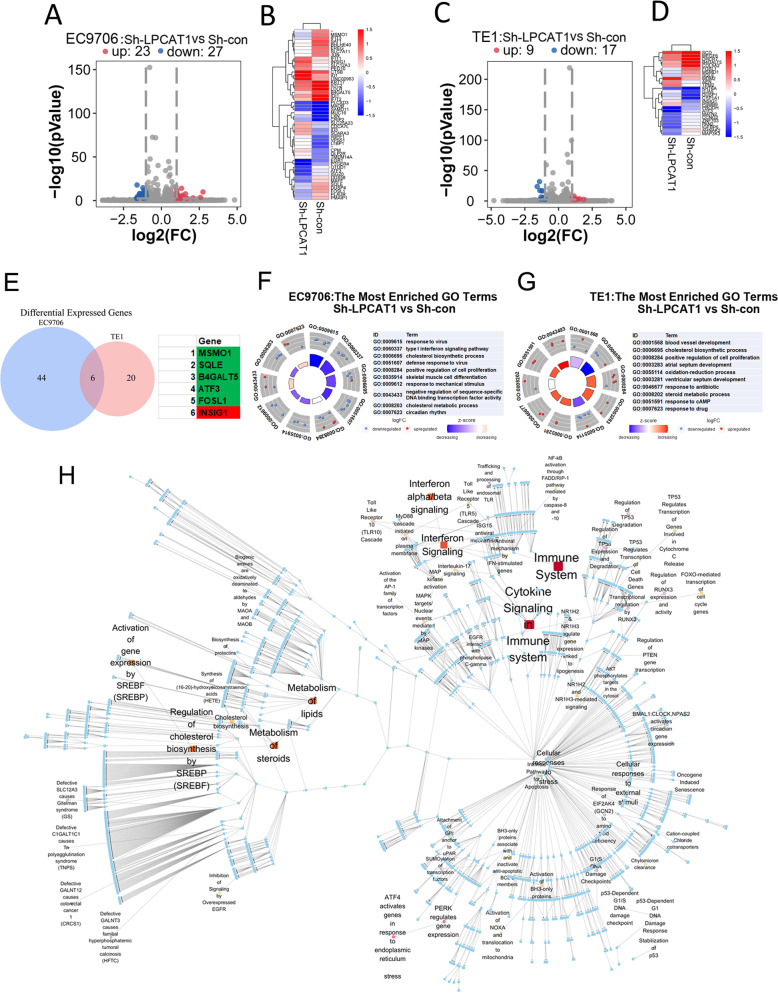
LPCAT1 promote the expression of cholesterol synthesis signal pathway genes in esophageal squamous cell carcinoma. **A** Volcano plots showing that, among differentially expressed genes, 23 were upregulated and 27 were downregulated in EC9706 cells after knockdown of LPCAT1. **B** A cluster heat map was used to show the expression variations of these gene transcripts in EC9706 cells. **C** Volcano plots illustrating that, among differentially expressed genes, 9 were upregulated and 17 were downregulated in TE1 cells after knockdown of LPCAT1. **D** A cluster heat map was used to show the expression variations of these gene transcripts in TE1 cells. **E** Venn analysis of genes with altered expression in EC9706 and TE1 cells after knockdown of LPCAT1. **F**, **G** All of the genes altered in EC9706 and TE1 cells were subject to GO analysis. **H** All genes with altered expression were enriched and analyzed by human gene signaling pathway.

Several metabolic mechanisms have been reported to be involved in tumor metastasis, but the characterization of the mechanisms regulated by LPCAT1 in ESCC cells is limited. To address this limitation, we compared EC9706 and TE1 cells transfected with or without LPCAT1 knockdown constructs using RNA sequencing analysis. We found that the expression of 50 genes in EC9706 cells and 26 genes in TE1 cells was altered after LPCAT1 knockdown (Fig. 4A–D). Finally, we identified six genes with altered expression in both EC9706 and TE1 cells (Fig. 4E). The GO enrichment analysis showed that MSMO1, SQLE, and INSIG-1 participate in the cholesterol biosynthetic process both in EC9706 and TE1 cells (Fig. 4F, G). To explore the potential pathway of LPCAT1 in ESCC in detail, genes influenced by LPCAT1 were clustered in Reactome database and were visualized on a local whole-human pathway network, which showed that cholesterol biosynthesis pathways were altered after LPCAT1 knockdown (Fig. 4H). These results indicate that MSMO1, SQLE, and INSIG-1, which are involved in the cholesterol biosynthesis pathway, may be regulated by LPCAT1 during ESCC development.

### LPCAT1 promotes esophageal tumor cholesterol synthesis through the EGFR/INSIG-1/SREBP-1 and phosphoinositide-3 kinase (PI3K)/SQLE pathways

**Fig. 5:**
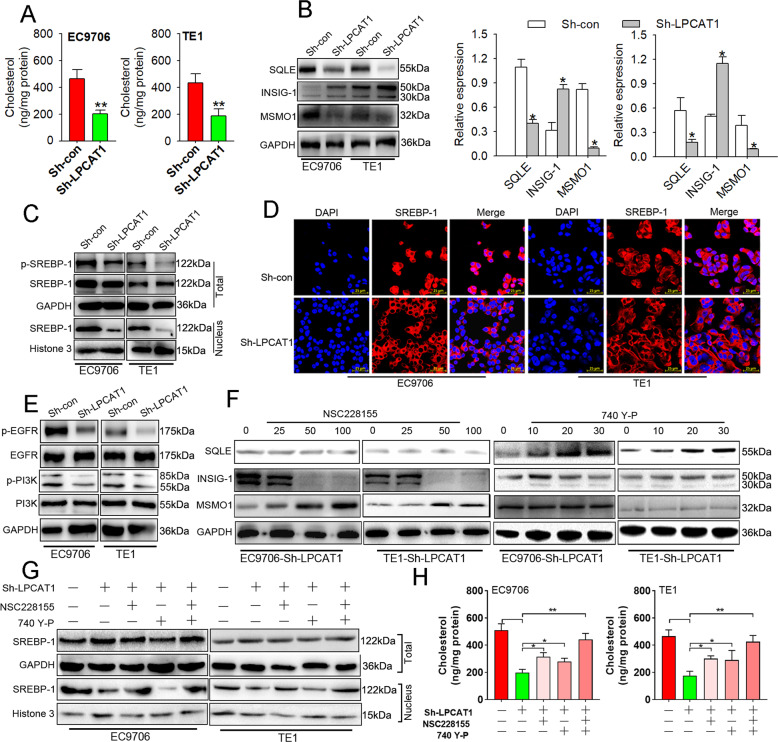
LPCAT1 promote ESCC cholesterol synthesis by EGFR/INSIG-1/SREBP-1 pathway. **A** The level of cholesterol in EC9706 and TE1 cells transfected with sh-control and sh-LPCAT1 was detected using the cholesterol detection kit. **B** The expression of SQLE, Insig-1, and MSMO1 in EC9706 and TE1 cells transfected with sh-control and sh-LPCAT1 was detected using western blot. **C**, **D** The expression of p-SREBP-1 in the nucleus in EC9706 and TE1 cells transfected with sh-control and sh-LPCAT1 was detected using western blot and immunofluorescence. **E** The expression of p-EGFR and p-PI3K in EC9706 and TE1 cells transfected with sh-control and sh-LPCAT1 was detected using western blot. **F** The expression of SQLE, Insig-1, and MSMO1 in EC9706 and TE1 cells treated with NSC228155 or 740Y-P after transfection with sh-LPCAT1 was detected using western blot. **G** The expression of p-SREBP-1 in the nucleus in EC9706 and TE1 cells treated with NSC228155 or 740Y-P after transfection with or without sh-LPCAT1 was detected using western blot. **H** The level of cholesterol in EC9706 and TE1 cells treated with NSC228155 or 740Y-P after transfection with or without sh-LPCAT1 was detected using the cholesterol detection kit. Data represent three independent experiments. *P < 0.05, **P < 0.01 (unpaired *t* test, one-way ANOVA). Data are from three independent experiments.

To further explore the mechanism underlying the involvement of LPCAT1 in ESCC development, we assessed the levels of cholesterol in ESCC cells and observed a reduction in cholesterol synthesis (Fig. 5A). Furthermore, the expression of PC was reduced while LPC was upregulated after knocking down LPCAT1 in ESCC cells (Supplementary Fig. 4). We found that the loss of LPCAT1 resulted in the downregulation of SQLE and MSMO1 and upregulation of INSIG-1 in vitro (Fig. 5B), suggesting that the cholesterol metabolism pathway is involved in the inhibition of ESCC development. In addition, analysis of the clinical tumor samples also showed that the expression of MSMO1 and SQLE was much higher in the tumor tissues compared to that in normal healthy controls and was positively correlated with the expression of LPCAT1 (Supplementary Figs. 5 and 6), while the expression of INSIG-1 showed the opposite results (Supplementary Fig. 7).

The nuclear form of SREBP binds to the sterol regulatory element (SRE) and enhances the target gene expression through which the lipid biosynthesis is increased [25]. Hence, we showed that the phosphorylation of SREBP-1 was decreased after LPCAT1 knockdown (Fig. 5C, D and Supplementary Fig. 8A, B). SREBPs are located in the endoplasmic reticulum (ER) membrane complexed with the SREBP cleavage activating protein (SCAP) in a tight association with insulin-induced gene (INSIG), which retains SREBP when cellular sterol levels are sufficient. In addition to regulation by sterols, SREBP-1 has been shown to be stabilized and activated by the PI3K/Akt oncogenic signaling pathway in cancer [26]; hence, we assessed the activation of EGFR and PI3K in ESCC cells and showed that the phosphorylation of EGFR and PI3K was significantly inhibited following LPCAT1 knockdown (Fig. 5E and Supplementary Fig. 8C, D). Consistent with these results, we also found that the expression of INSIG-1 decreased while that of MSMO1 was increased after treatment with NSC228155, an agonist of EGFR, but no changes were observed after treatment with 740 Y-P, an agonist of PI3K (Fig. 5F and Supplementary Fig. 8F, G, I, J). Interestingly, we found that SQLE, which is a cholesterol synthesis enzyme, was increased after treated with 740 Y-P (Fig. 5F and Supplementary Fig. 8E, H). In addition, the phosphorylation of SREBP-1 was also reversed after activation of EGFR but not PI3K following LPCAT1 knockdown (Fig. 5G and Supplementary Fig. 8K-L). We also observed that the expression of SREBP-1 in the nucleus of tumor tissues with high LPCAT1 expression is significantly higher than that in the nucleus of tumor tissues with low LPCAT1 expression (Supplementary Fig. 9). Intriguingly, biosynthesis of cholesterol was also reversed following treatment with the agonists of EGFR and PI3K (Fig. 5H). In addition, the expression of c-myc, which is upregulated by LPCAT1 in patients with lung adenocarcinoma [27], was not enhanced compared with EGFR/PI3K in ESCC cells (Supplementary Fig. 10), which suggests that LPCAT1 may regulate cholesterol synthesis in ESCC cells primarily through these pathways. The activation of EGFR leads to the upregulation of MSMO1 and downregulation of INSIG-1; subsequently, SREBP-1 enters the nucleus after phosphorylation, and cholesterol synthesis increases. At the same time, the activation of PI3K leads to the upregulation of SQLE, which accelerates the synthesis of cholesterol.

### Activated PI3K upregulates the transcriptional activity of SQLE via the transcription factors SP1 and SREBF2

**Fig. 6:**
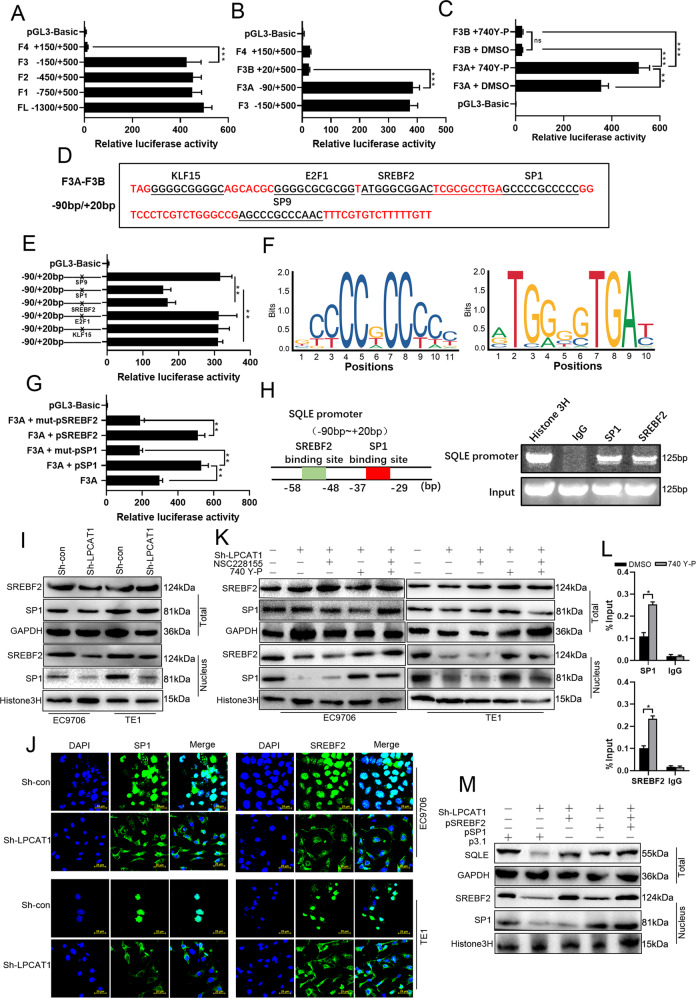
PI3K signaling promotes cholesterol synthesis by upregulation of SQLE transcriptional activity via SP1 and SREBF2. **A**, **B** To explore the key transcriptional activity region, serial truncated plasmids of the SQLE promoter and the pRL-TK plasmid were transfected into TE1 cells for a dual-luciferase reporter assay. **C** DMSO and 20 μM 740Y-P were added after SQLE-truncated plasmids and the control plasmid were co-transfected and the promoter activity was measured with the dual-luciferase reporter assay. **D** The transcription factor-binding sites on the 5‘-regulatory region sequence of SQLE were predicted using the JASPAR online software. **E** The mutant plasmids were transiently transfected into HeLa cells and the luciferase activity was measured after 48 h. **F** The colorful letters indicate the motif of transcription factor-binding site of SP1 and SREBF2 cited from JASPAR. **G** The transcriptional activity of SQLE was elevated after overexpression of SP1 and SREBF2. **H** Chip-PCR detection of SP1 and SREBF2 binding in the promoter of SQLE. **I**, **J** The expression levels of SP1 and SREBF2 in nuclear was detected using western blot (**I**) and immunofluorescence (**J**) after knocking down of LPCAT1. **K** The expression of SREBF2 and SP1 in EC9706 and TE1 cells treated with NSC228155 or 740Y-P after transfection with or without sh-LPCAT1 was detected using western blot. **L** TE1 cells treated with the PI3K agonist (20 μM) or DMSO were subjected to a ChIP assay. IgG was served as a negative control. The results from three independent experiments. **M** The expression of SQLE and nucleus SP1 and SREBF2 after overexpression of SP1 and SREBF2 in TE1 cells that were transfected with or without sh-LPCAT1 was detected using western blot. Normalized luciferase activity and *P* values are from three independent experiments. *P < 0.05, **P < 0.01, ***P < 0.001 (unpaired *t* test, one-way ANOVA). Data are from three independent experiments.

In order to investigate the regulation of SQLE expression after PI3K activation, we analyzed the transcriptional regulatory region of the human SQLE promoter. The dual-luciferase reporter assay results suggested that the key region bearing promoter activity of SQLE was located in F3A to F3B (−90 bp/+20 bp; Fig. 6A, B). To further clarify whether PI3K activation was responsible for the upregulated expression of SQLE, we used a PI3K activator after transfection of truncated plasmids and found that the activator was able to significantly increase the transcriptional activity of SQLE (Fig. 6C). We next predicted and analyzed transcription factors that might bind to the key region bearing promoter activity of SQLE using bioinformatics (Fig. 6D). We constructed transcription factor-binding site mutant plasmids and transfected them into ESCC cells to detect the transcriptional activity. The results showed that the promoter activity of SQLE decreased 50–60% when the binding sites of SP1 and SREBF2 were mutated (Fig. 6E). Furthermore, we overexpressed the normal and mutant SP1 and SREBF2 in F3A-transfected ESCC cells separately. As shown in Fig. 6F, G, both SP1 and SREBF2 can elevate the transcriptional activity of SQLE. Chromatin immunoprecipitation (ChIP) results indicated that both the transcription factors, SP1 and SREBF2, interacted with the promoter of SQLE (Fig. 6H and Supplementary Fig. 11A).

To further clarify whether the expression of LPCAT1 can regulate the transcription factors SP1 and SREBF2, we detected the expression of SP1 and SREBF2. As shown in Fig. 6I, J and Supplementary Fig. 11B–E, LPCAT1 promoted the entry of SP1 and SREBF2 into the nucleus (Fig. 6I, J and Supplementary Fig. 11B–E). In addition, we determined whether the increase of SP1 and SREBF2 in the nucleus and recruitment of SP1 and SREBF2 to SQLE promoter was in response to the activation of PI3K (Fig. 6K, L and Supplementary Fig. 11F–I). To further confirm that the increase of SP1 and SREBF2 in the nucleus can upregulate the transcriptional activity of SQLE, we overexpressed SP1 and SREBF2 in TE1 cells (Supplementary Fig. 12). The overexpression of SP1 and SREBF2 resulted in the reversal of the downregulation of SQLE caused by LPCAT1 knockdown (Fig. 6M and Supplementary Fig. 11J–L). Together, these data demonstrate that LPCAT1/PI3K signaling pathway regulates the expression of SQLE by regulating the incorporation of SP1 and SREBP-1 into the nucleus.

### LPCAT1 promotes ESCC development and cholesterol synthesis in vivo

**Fig. 7:**
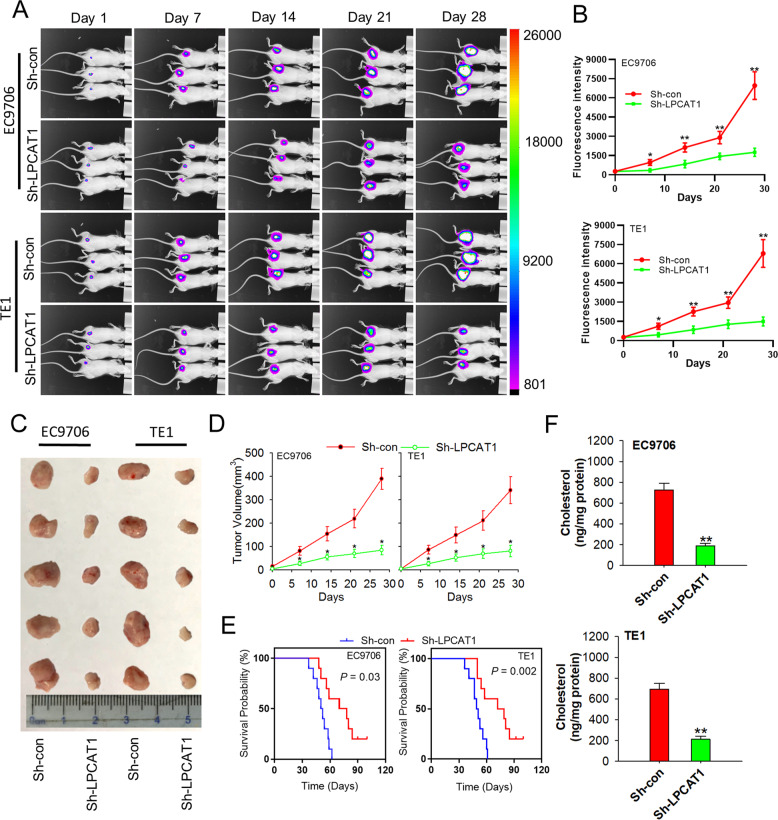
LPCAT1 promote ESCC development and cholesterol synthesis in vivo. EC9706 and TE1 cells stably transfected with sh-control and sh-LPCAT1 were injected subcutaneously into NOD/SCID mice. Four weeks after the injection, mice were sacrificed by carbon dioxide suffocation. **A** Representative bioluminescent/photographic images in the nude mouse. **B** Quantitation of signals plotted against time points. **C** Representative images of tumor nodes were shown. **D**, **E** Tumor growth (**D**) and survival curves (**E**) are plotted. **F** Cholesterol was detected using the cholesterol detection kit in the tumor tissues of each group. Data are from three independent experiments. *P < 0.05, **P < 0.01 (unpaired *t* test). Data are from three independent experiments.

To further investigate whether LPCAT1 contributes to tumor progression in vivo, ESCC cells with or without LPCAT1 depletion using lentivirus were injected into the flank of nude mice. As shown in Fig. 7A–D, when compared to the control, tumors derived from LPCAT1-depleted cells were smaller and had a reduced growth rate (Fig. 7D). In line with this, using the Kaplan–Meier survival curves and log-rank test, differences were found in nude mice inoculated with ESCC cells with or without LPCAT1 depletion (Fig. 7E). Additionally, the level of cholesterol was much lower in mice injected with ESCC cells with LPCAT1 depletion than those injected with ESCC control cells (Fig. 7F). These results demonstrate that LPCAT1 promotes ESCC development and cholesterol synthesis in vivo.

## Discussion

Although the mechanisms and biogenesis of tumor development have been intensively investigated in recent years, the metabolic functions of cancer are still not well understood. In this study, we found that the expression of the LPCAT1 enzyme is enriched in esophageal cancer. Overexpression of LPCAT1 in ESCC potentiates multiple tumor characteristics, including cell migration and invasion [16]. These functions greatly contribute to cancer metastasis and malignancy, which further support our observations that high levels of LPCAT1 are associated with poor prognosis in ESCC patients. Although several oncogenes that play critical roles in the development of ESCC have been identified, the role of cholesterol metabolism, especially the enzymes related to its metabolic regulation, in ESCC metastasis is largely unknown. To our knowledge, this is the first report that thoroughly investigates the expression, function, mechanism, and clinical implications of LPCAT1 in ESCC.

Previous studies have shown that some enzymes related to cholesterol metabolism play special roles in tumor biology [28, 29]. Other studies have found that enzymes related to metabolism, which control specific lipid properties of the cell membrane, are required for oncogenic receptors to properly localize on the cell surface [30, 31]. Targeting the enzyme that controls specific lipid properties of the cell membrane resulted in the dissociation of oncogenic growth factor receptors from the cell membrane, which blocked their signaling and induced significant tumor cell death and greatly increased survival in mice bearing a variety of different tumors [32, 33]. A previous study highlighted an important link between cancer-causing genes and membrane lipids, suggesting that these metabolic genes can serve as new cancer drug targets [34]. Besides, upregulation of *LPCAT1* has been reported in multiple cancers and links phospholipid changes to cancer cell proliferation and invasion ability [15, 33, 35, 36]. However, the mechanism by which LPCAT1 regulates esophageal cancer has remained largely unknown. In this study, we found that LPCAT1 promoted the development of esophageal cancer. LPCAT1 was highly expressed in esophageal cancer tissues and promoted esophageal cell invasion and migration via the SREBP-1/EGFR/PI3K signaling pathway. We also found that, following LPCAT1 knockdown, the phosphorylation of EGFR was downregulated while that of INSIG-1 was upregulated. The upregulation of INSIG-1 prohibits the phosphorylation of SREBP-1, which blocks SREBP-1 translocation into the nucleus, thereby prohibiting the synthesis of cholesterol. We found that the expression of INSIG-1 was upregulated, but the expression of the cholesterol synthesis rate-limiting enzyme, SQLE, was not affected following treatment with NSC228155, an agonist of EGFR. Intriguingly, the activation of PI3K leads to the upregulation of SQLE, which accelerates the synthesis of cholesterol. Therefore, our study suggests that LPCAT1 may reregulate cholesterol synthesis in ESCC mainly through these two pathways.

In esophageal cancer, LPCAT1 induces esophageal tumor development by influencing cholesterol metabolism through a mechanism that is different from those previously identified in esophageal tumor research. As SREBPs are major transcription factors activating the expression of genes involved in the biosynthesis of cholesterol, fatty acids, and triglycerides, inhibition of SREBP can decrease the biosynthesis of cholesterol and fatty acids [37, 38]. The nuclear form of SREBP binds to SRE and enhances the target gene expression through which the lipid biosynthesis is increased [25]. SREBPs are located in the ER membrane complexed with SCAP in tight association with INSIG, which retains SREBP in the ER when cellular sterol levels are sufficient. However, in cancer cells, SREBP-1 regulation appears to be more complex. Our work suggests that LPCAT1 can affect cellular metastasis and regulate signal transduction pathways involved in the process, suggesting a novel role for LPCAT1 in esophageal cancer progression. EGFR/INSIG-1/SREBP-1 signaling is critical for tumor growth, maintenance, and metastasis.

SQLE is an enzyme that converts squalene to 2,3-oxidosqualene in the early stage of cholesterol generation. Interestingly, our results indicated that PI3K upregulated the transcriptional activity of SQLE. Cancer cells require high levels of cholesterol to rapidly form new membranes for division [39]. However, the mechanisms through which cancer cells maintain their cholesterol levels are not completely known. Exogenous uptake and de novo synthesis both contribute to cellular cholesterol pools [40], but the preferred route in cancer cells is unclear. However, our study showed that SP1 and SREBF2 can elevate the transcriptional activity of SQLE, and the ChIP results indicated that both these transcription factors interacted with the promoter of SQLE in ESCC cells. Together, these data demonstrate that LPCAT1/PI3K signaling pathway regulates the expression of SQLE by regulating the incorporation of SP1 and SREBP-1 into the nucleus. Therefore, LPCAT1 could be a potential therapeutic target for cancer.

Virtually all these molecules that are implicated in the regulation of processes that control cell growth and proliferation depend on the sufficient supply of lipids and fatty acids, some of which are known to directly contribute to the rate-limiting steps of lipid metabolism [41, 42]. Clinical data show that the 5-year survival rate in early-stage ESCC patients after surgery is much higher than that in late-stage ESCC patients [43]. However, ESCC is associated with symptoms that do not enable early diagnosis, and therefore, the majority of ESCC patients are diagnosed at advanced stages when the best therapeutic window for treatment is lost [44, 45]. Currently, traditional tumor markers, such as carcinoembryonic antigen (CEA) and cytokeratin 19 fragment (CYFRA 21-1), are used to diagnose and evaluate ESCC progression but their sensitivity and validity are insufficient for early ESCC detection [44]. Therefore, improved biomarkers that allow early ESCC detection are urgently needed. Metabolic disorders have been recently identified as new factors that can affect tumor development. A growing number of studies have demonstrated that LPCAT1 plays a significant role in cancer pathogenesis [46]. In this study, we demonstrated the potential of LPCAT1 as a new prognostic marker.

This study has some limitations. Whether LPCAT1 combined with CEA could serve as an effective biomarker for ESCC remains to be determined using a comprehensive large cohort study for ESCC diagnosis and treatment.

In summary, our findings show that LPCAT1 is an upstream regulator of the EGFR/INSIG-1/SREBP-1 and PI3K/SP1/SREBF2 signaling pathways in ESCC cells. The overexpression of *LPCAT1* is associated with lymph node metastasis and poor prognosis in ESCC patients. Our in vivo experiments and the clinical characteristics of LPCAT1 expression indicate that inhibition of *LPCAT1* may have therapeutic value for ESCC treatment and that LPCAT1 has the potential to serve as a biomarker for ESCC diagnosis and prognosis.

## Materials and methods

### Tissue and serum samples

Tumor tissues and adjacent normal esophageal tissues were collected from 154 patients with ESCC who underwent radical surgery of ESCC at the Department of Thoracic Surgery, the Affiliated Huaian No.1 People’s Hospital of Nanjing Medical University from December 2012 to November 2013. Blood samples from healthy individuals were collected form Health Examination Centers. All samples were stored in ESCC biological sample bank and collected under the guidance of the HIPAA protocol and supervised by the ethics committee of Huaian No.1 People’s Hospital. All patients were diagnosed as ESCC by pathology after endoscopic biopsy before operation and provided written informed consent before inclusion in this study. Tumor, Node, Metastasis (TNM) stage classification complied with the TNM classification system of the International Union Against Cancer. We used Kaplan–Meier method to draw the overall survival curve according to the relative expression of LPCAT1 and the cut-off value for LPCAT1. This study was approved by the ethics committee of Affiliated Huaian No.1 People’s Hospital, Nanjing Medical University (YX-P-2020-055-01).

### Quantitative proteomics using liquid chromatography tandem mass spectrometry (LC-MS/MS)

Proteins were extracted using the protein extraction kit (Thermo Fisher, CA, USA). The DDA spectrum library was constructed. LC-MS/MS was used for DDA mode and DIA mode analysis. DDA and DIA data were analyzed using Proteome Discoverer 2.2 (PD 2.2, Thermo) platform, Biognosys Spectronaut version 9.0, and R statistical framework. DDA MS raw files were analyzed using the PD software (version 2.2) and peak lists were searched against protein database. The MS proteomics data have been deposited to the ProteomeXchange Consortium (http://proteomecentral.proteomexchange.org) via the iProX partner repository with the dataset identifier PXD020230.

### Cell culture

HEEC, EC9706, and TE1 cell lines were purchased from the Shanghai Cell Bank Type Culture Collection Committee (CBTCCC, Shanghai, China). HEEC and TE1 cell lines were cultured in RPMI-1640 containing 10% fetal bovine serum (FBS) and 1% penicillin–streptomycin (PS). EC9706 cell lines were maintained in Dulbecco’s Modified Eagle Medium (DMEM) supplemented with 10% FBS and 1% PS. All cells were grown at 37 °C and 5% CO_2_ in a humidified incubator (Thermo Fisher, USA). All cell lines were verified using short tandem repeat analysis.

### RNA isolation and quantitative real-time PCR (qRT-PCR)

Total RNA was extracted from tissue samples or cells with TRIzol reagent (Thermo Fisher, CA, USA). The concentration and purity of RNA were determined using a NanoDrop 2000 (Thermo Fisher, CA, USA). First-strand cDNA was synthesized using a reverse transcription reagent kit (TaKaRa Bio, Japan) according to the manufacturer’s instructions. Quantitative real-time reverse transcription PCRs were performed on a LightCycler 480 Real-time PCR System Roche, Shanghai, China) with Universal SYBR Green Master Mix (TOYOBO life science, Shanghai, China). The specific primers used are provided in Supplementary Table 1. *GAPDH* served as the internal references. After normalizing the CT value, the relative of gene expression was calculated using the 2^−ΔΔCt^ method [47]. Each sample tested was from three independent experiments.

### Immunoblotting

Equal amounts of 0.2 g tissue and counted cells were homogenized in a total of 2 ml RIPA (Beyotime, Beijing, China) buffer containing 1% phenylmethanesulfonylfluoride (Thermo Fisher, CA, USA) using a DHS Q24R homogenizer (Beyotime, Beijing, China). The homogenate was then centrifuged at 12,000 rpm for 20 min at 4 °C, and the supernatant proteins were collected. Protein concentration was determined using the BCA method (Biosharp, Shanghai, China). After denaturation at 100 °C for 10 min, proteins were separated using sodium dodecyl sulfate-polyacrylamide gel electrophoresis and transferred onto polyvinylidene difluoride membranes (Millipore, Bedford, MA). The membranes were blocked with 5% bovine serum albumin (BSA; Thermo Fisher, CA, USA) in TBST buffer at room temperature for 2 h, then incubated overnight at 4 °C with the primary antibody. We used a corresponding horseradish peroxidase (HRP)-labeled secondary antibody (Thermo Fisher, CA, USA) to incubate the membrane for 2 h at room temperature and then washed 3 times with TBST buffer. The proteins were visualized using an enhanced chemiluminescent (Thermo Fisher, CA, USA) detection reagent (Tanon, Shanghai, China).

### IHC analysis

The ESCC tissues were fixed with 10% formalin and embedded in paraffin for tissue microarray before the sections were treated with specific primary antibodies. After antigen repair, the sections were blocked with 1% BSA (Thermo Fisher, CA, USA) in PBST buffer at room temperature for 2 h, then incubated at 4 °C overnight with the primary antibody. The sections were washed three times and incubated with HRP-polymer-conjugated secondary antibody (Thermo Fisher, CA, USA) at room temperature. Then the sections were stained with 3,3-diaminodbenzidine substrate and hematoxylin. The slides were scanned using pathological imaging system. The IndicaLabs HALO^TM^ software was used for quantitative analysis.

### immunofluorescence analysis

The ESCC cell lines were seeded in chamber slide (NUNC Lab-Tek^TM^, Denmark) and incubated in RPMI 1640 or DMEM medium at 37 °C in a humidified atmosphere of 5% CO_2_ for 1 night. The ESCC tissues were fixed as previously described [47] and embedded in tissue-freezing medium (Leica, Germany) and stored at −20 °C. Tissue sections of 5 μm were made with freezing microtome (Leica, Germany). The immunofluorescence staining for ESCC cells and tissue were performed as previously described [47]. Samples were observed using a confocal microscope (Nikon, Japan).

### Transient transfection and establishment of stable cell lines

For transient transfection of ESCC cell lines, si-LPCAT1 RNAs and negative control RNAs were synthesized (GenePharma, Shanghai, China) and transfected into cells using the Lipofectamine 2000 Kit (Thermo Fisher, CA, USA) according to the manufacturer’s instructions. The sequences of siRNA are listed in Supplementary Table 2. The cells were harvested for western blot after a 48-h transfection. To produce cell lines with a stable expression of the LPCAT1 knockdown construct, TE1 and EC9706 cells were infected with shLPCAT1 or shNC viruses. The shLPCAT1 lentiviral particles was prepared by selecting the siRNA sequence—from among two si-LPCAT1 RNAs—that could efficiently knock down *LPCAT1* expression. After selection with puromycin, cells with stable transfection were used for further experiments.

### Cell Counting Kit-8 (CCK8) assays

Transient transfection of ESCC cells were seeded in 96-well flat-bottomed plates with each well containing 2000 cells in 100 μl of culture medium. A volume of 10 μl CCK-8 solution was added to each well and incubated at 37 °C for 2 h at 24, 48, and 72 h. The absorbance at 450 nm of the experimental wells were measured using an automatic microplate reader (Tecan, Switzerland).

### Clonogenic assay and soft agar assay

For the clonogenic assay, 500 cells were seeded in a 6-well plate for 2 weeks. Cell colonies were fixed with 4% paraformaldehyde and stained with 0.1% crystal violet, and the stained colonies were imaged and then manually counted. Soft agar assay was performed as previously described [47]. After 21-day culture, the colonies of tumor cells were imaged and counted. The size of 20 randomly selected colonies per well was measured and calculated using the formula: (length + width)/2.

### Transwell assay

Transwell assay was used for the migration and invasion assessments. A total of 1 × 10^5^ cells were resuspended in 200 μl DMEM without FBS and seeded in the upper chamber of a Transwell insert in a 24-well plate (NEST, WuXi, China). The lower chambers contained DMEM with 20% FBS. After 24 and 48 h incubation, cells on the bottom surface of the membrane were fixed and stained with 0.1% crystal violet. Meanwhile, cells on the upper surface of the inserts were then removed by scraping the inserts with a cotton swab. Migration was assessed by counting the number of penetrated cells in five random fields. Matrigel invasion assays were performed using Matrigel-coated Transwell inserts following the same procedure described above [48].

### Cell cycle and apoptosis assays

For cell cycle assays, ESCC cells were harvested 24 h after serum starvation and fixed in 80% ice-cold ethanol in phosphate-buffered saline (PBS) after washing in ice-cold PBS. Then cells were incubated at 37 °C for 30 min; bovine pancreatic RNAase (Sigma) was added at a final concentration of 2 mg/ml and 20 mg/ml of PI (Sigma-Aldrich, USA) for 30 min at room temperature. Cell cycle distribution was flow cytometrically determined using a FACScan (Becton Dickinson, Franklin Lakes, NJ, USA). For apoptosis assays, a fluorescein isothiocyanate (FITC) Annexin V Apoptosis Detection Kit (Dojindo, Kumamoto, Japan) was used according to the manufacturer’s instructions. Briefly, cells were collected by mild trypsinization, washed twice in cold PBS, stained with FITC-Annexin V and PI on ice for 5 min, and subjected to flow cytometry using a FACScan (Becton Dickinson, Franklin Lakes, NJ, USA).

### RNA sequencing

After total RNA was extracted, mRNA was isolated with oligo magnetic beads and cut into small fragments for cDNA synthesis. Libraries were generated using the NEBNext Ultra^TM^ RNA Library Prep Kit (New England Biolabs, Ipswich, MA, USA) for the Illumina system according to the manufacturer’s instructions. Sequencing was conducted using the Illumina Hiseq XTEN platform.

### Pathway mapping

Pathway relationship file were download from Reactome [49] and the whole human pathway network was reconstructed locally using Cytoscape 3.8 [50]. Upregulated and downregulated genes were clustered in Reactome and were matched on the reconstructed pathway network.

### Plasmid construction

The human SQLE promoter (from −1300 to +500 bp, located in the upstream and downstream of the transcriptional start site), were obtained from the National Center for Biotechnology Information (http://www.ncbi.nlm.nih.gov/). Potential transcription factor-binding sites were predicted using the JASPAR network tool software (http://jaspar.genereg.net/). The fragments were cloned into pGL3-Basic vectors (Promega, USA) and renamed pGL3-SQLE-FL. In addition, successive deletions of the SQLE promoter was performed as previously described [51]. The coding regions of Sp1 and SREBF2 were cloned into pcDNA3.1^+^ plasmid as previously described [47]. The primers used in this study are listed in Supplementary Table S3. Plasmids were extracted using an Endo-free Plasmid Mini Kit II (Omega Bio-Tek, USA). To define the binding sites of transcription factors on the promoter of the SQLE gene, site mutation of the human SQLE promoter were constructed by PCR amplification using pGL3-SQLE-F3A as backbones, and pRL-TK plasmids were co-transfected as an internal control in dual-luciferase reporter assay. The primers are listed in Supplementary Table 4. Transfection and dual-luciferase reporter assay were performed as previously described [52].

### ChIP assay

ChIP analysis was performed according to the instruction of ChIP Assay Kit (56383, Cell Signalling Technology, America). TE1 cells were seeded in 6-well and fixed by adding 16% formaldehyde directly into the culture media after culturing for 48 h. After 10 min fixing in room temperature, glycine was added to the media to stop fixing. Chromatin was fragmented to 200–400 bp using 20 cycles (30 s on, 30 s off, High Setting) by sonication. ChIP was conducted overnight at 4 °C with the primary antibodies Histone H3 (4620, Cell Signalling Technology, America, provided in the ChIP Assay Kit), SP1 (Proteintech, America), SREBF2 (Proteintech, America), and 2 μl of the normal Rabbit IgG (provided in the ChIP Assay Kit), respectively. After reverse cross-linking and DNA extraction, immunoprecipitated chromatin was used as the template for RT-PCR analysis. The primers for detection of the promoter of SQLE is listed in Supplementary Table 3.

### In vivo xenograft experiments

Six-week-old SCID/NOD mice were purchased from Jiangsu ALF Biotechnology Co., LTD (Nanjing, China). EC9706 and TE-1 cells (1 × 10^7^) transfected with lentivirus containing sh-LPCAT1 or sh-con were subcutaneously injected into the armpits of the mice. Tumor size and survival rate were measured every 7 days. On day 28, 5 mice each group were euthanized, and tumor tissues were harvested for cholesterol detection using a cholesterol detection kit (Biovision, USA). The survival time of each group of mice was recorded in another repeat experiment. This study was approved by the Ethics committee of Huai’an No. 1 People’s Hospital and was conducted in accordance with the guidelines of the National Animal Care and Ethics Institution [53].

### Reagents and antibodies

Regarding the primary antibodies used in the study, anti-GAPDH (60004), anti-MYC(10828-1-AP), anti-LPCAT1 (66044, Mouse Monoclonal), and anti-SREBP1 (14088) were purchased from Proteintech (IL, USA); anti-MSMO1 (Thermo Fisher, USA), anti-SQLE, anti-LPCAT1 (ab214034, Rabbit Monoclonal), and anti-INSIG1 (ab70784, Rabbit polyclonal) were purchased from Abcam (Cambridge, UK); anti-phospho-SREBP1 (AF3283) was purchased from Affinity (OH, USA); anti-EGFR (#4267S), anti-phosphor-EGFR (#3777S), anti-PI3K (#4228), and anti-phosphor-PI3K (#4292) were purchased from Cell Signaling Technology (Danvers, PA USA). The restriction endonucleases (*KpnI*, *Hind III*, *Nhe I*, *Xba I*), KOD FX Neo were purchased from TOYOBO (SHANGHAI) BIOTECH CO., LTD. The Seamless Assembly Cloning Kit (CloneSmarter, USA) was purchased from Taihe Biotechnology Co., LTD. The activators 740 Y-P and NSC 228155 were purchased from MedChemExpress (NJ, USA).

### ELISA quantitation of LPCAT1

LPCAT1 in the serum of patients with ESCC was measured using an ELISA Kit (Reddot Biotech, RD-LPCAT1-Hu) according to the manufacturer’s instructions. Lysophosphotidylcholine and phosphatidylcholine in ESCC cells were also measured using ELISA Kits (Jiangsu Enzyme Industry Co., Ltd MM-50888H1 and MM-1693H1) according to the manufacturer’s instructions.

### Statistical analysis

Data were graphed using the GraphPad Prism software. Survival curves were estimated by Kaplan–Meier analysis and compared by the log-rank test. All results were confirmed in at least three independent experiments; Student’s *t* tests were used for between-group comparisons of the means of quantitative data, and *P* value <0.05 was considered statistically significant.

## Supplementary information

Supplemental Figure 1

Supplemental Figure 2

Supplemental Figure 3

Supplemental Figure 4

Supplemental Figure 5

Supplemental Figure 6

Supplemental Figure 7

Supplemental Figure 8

Supplemental Figure 9

Supplemental Figure 10

Supplemental Figure 11

Supplemental Figure 12

Supplemental tables

## Data Availability

All data, models, and code generated or used during the study appear in the submitted article.
