# Supplementary material for: LPCAT1 reprogramming cholesterol metabolism promotes the progression of esophageal squamous cell carcinoma
Source: Cell Death Dis. 2021 Sep 13;12(9):845. doi: 10.1038/s41419-021-04132-6 (PMC8438019; doi:10.1038/s41419-021-04132-6)
Supplement: Supplementary file 1 — Supplemental Figure 1 [file 41419_2021_4132_MOESM1_ESM.docx]

**
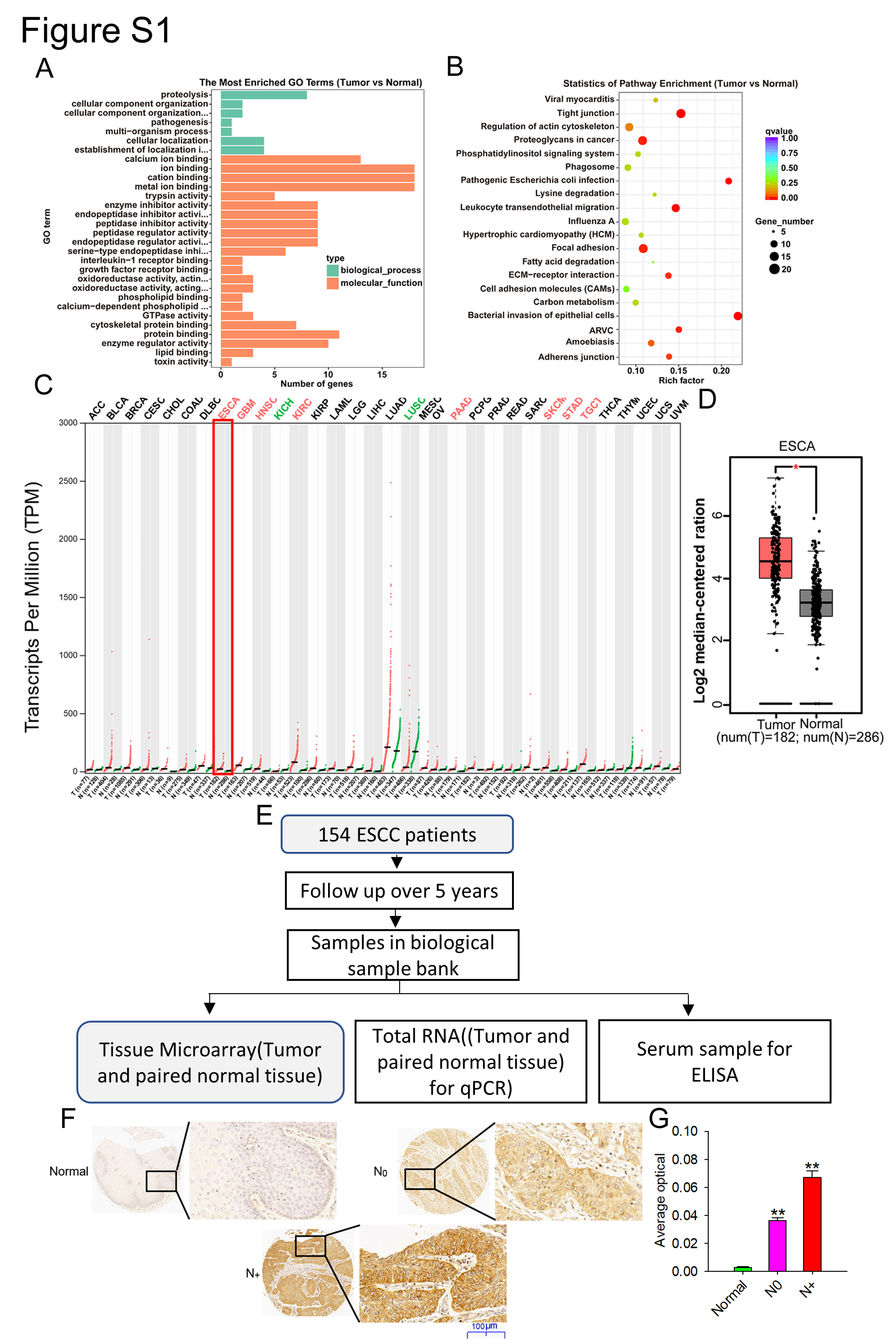
**

**Supplementary Figure 1. LPCAT1 is overexpressed in ESCC.**

**A-B.** Protein expression profile in ESCC tissues and their matched adjacent normal tissues using mass spectrographic analysis. All these genes were subject to GO term analysis (B). Enriched KEGG pathway analysis of the genes by DAVID (**B**). **C-D**. The copy number of LPCAT1 in esophageal cancer and 23 common tumors was directly proportional to the amount of mRNA expressed in the GEPIA database (<http://gepia.cancer-pku.cn/>). **E**. 154 ESCC samples which stored in Biological Sample Bank of esophageal cancer were involved for this research. **F-G.** The protein of LPCAT1 in 154 ESCC tissues in different lymph node stages was determined by immunohistochemistry analysis. Representativ IHC staining **(F)** and quantitative analysis of LPCAT1 **(G)**. Data are expressed as mean ± SD, *P < 0.05, **P < 0.01 (Unpaired *t*-test, one-way ANOVA).
