# Supplementary material for: LPCAT1 reprogramming cholesterol metabolism promotes the progression of esophageal squamous cell carcinoma
Source: Cell Death Dis. 2021 Sep 13;12(9):845. doi: 10.1038/s41419-021-04132-6 (PMC8438019; doi:10.1038/s41419-021-04132-6)
Supplement: Supplementary file 2 — Supplemental Figure 2 [file 41419_2021_4132_MOESM2_ESM.docx]

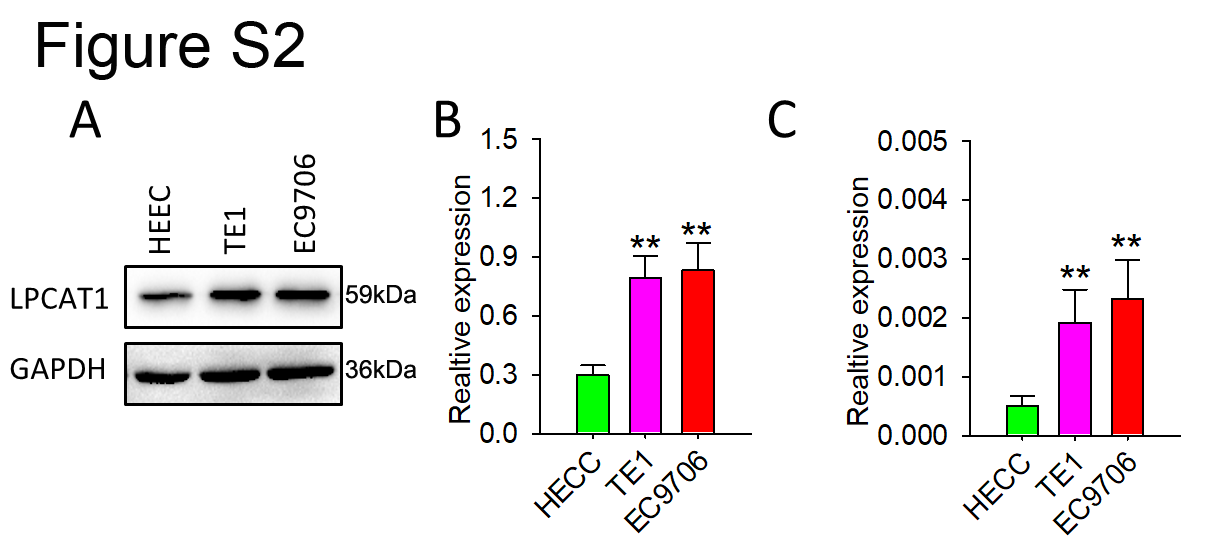


**Supplementary Figure 2. The expression of LPCAT1 was upregulated in ESCC cell lines.**

**A-B**. The expression of LPCAT1 protein in normal esophageal epithelial cells and esophageal squamous carcinoma cells. **C**. Gene expression of *LPCAT1* in normal esophageal epithelial cells and esophageal squamous carcinoma cells. Data are from three independent experiments. **P < 0.01 (one-way ANOVA).
