# Supplementary material for: LPCAT1 reprogramming cholesterol metabolism promotes the progression of esophageal squamous cell carcinoma
Source: Cell Death Dis. 2021 Sep 13;12(9):845. doi: 10.1038/s41419-021-04132-6 (PMC8438019; doi:10.1038/s41419-021-04132-6)
Supplement: Supplementary file 3 — Supplemental Figure 3 [file 41419_2021_4132_MOESM3_ESM.docx]

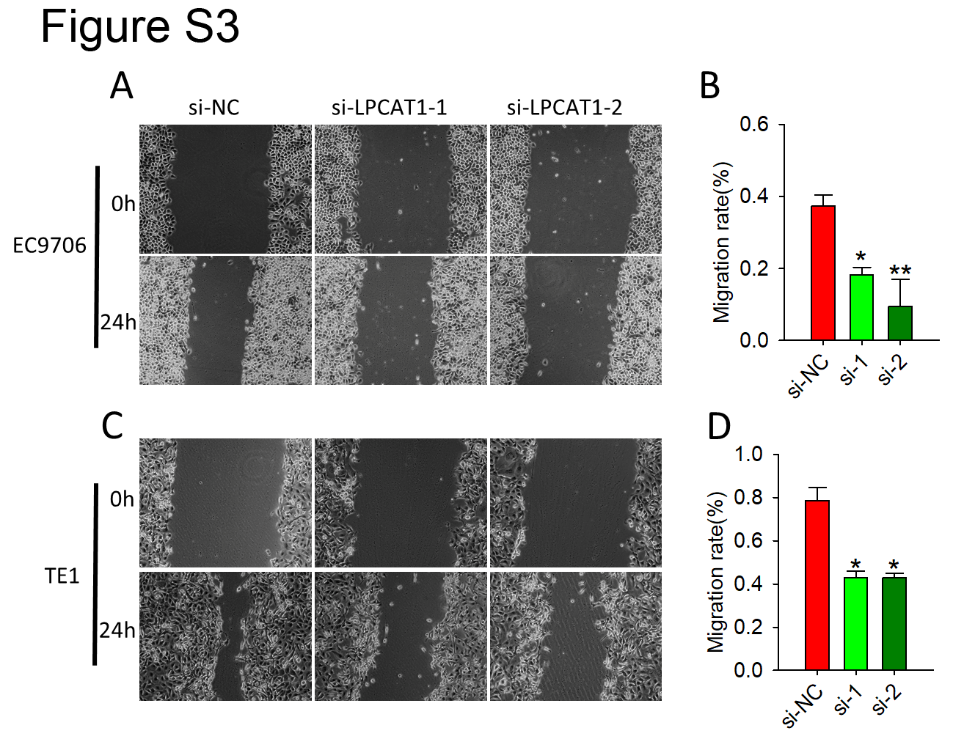


**Supplementary Figure 3. LPCAT1 promote ESCC migration.**

Wound-healing assays were performed to assess EC9706 cells (**A-B**) and TE1 cells (**C-D**) migration. Wound closure was determined 24 h after the scratch. Data are from three independent experiments and presented as mean ± SD, *P < 0.05, **P < 0.01 (one-way ANOVA).
