# Supplementary material for: LPCAT1 reprogramming cholesterol metabolism promotes the progression of esophageal squamous cell carcinoma
Source: Cell Death Dis. 2021 Sep 13;12(9):845. doi: 10.1038/s41419-021-04132-6 (PMC8438019; doi:10.1038/s41419-021-04132-6)
Supplement: Supplementary file 4 — Supplemental Figure 4 [file 41419_2021_4132_MOESM4_ESM.doc]

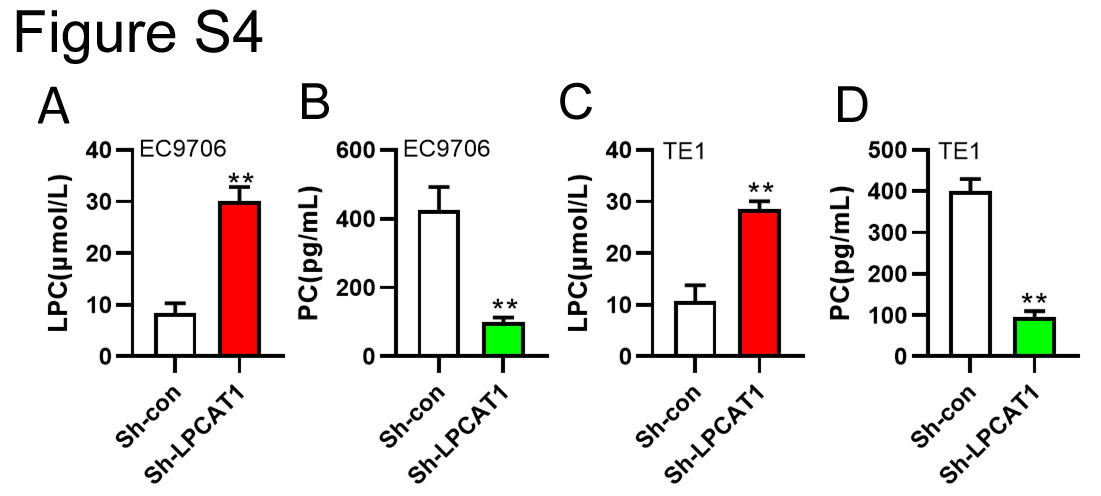


**Supplementary Figure 4. lysophosphotidylcholine (LPC) was up-regulated while Phosphatidylcholine (PC) was reduced after knocking down the LPCAT1** **in ESCC cells.**

Lysophosphotidylcholine (LPC)(A)(C) and Phosphatidylcholine (PC)(B)(D) were detected by the ELISA kit in ESCC cells transfected with sh-control and sh-LPCAT1, the results from three independent experiments. Data are from three independent experiments and presented as mean ± SD ***P* < 0.01, (Unpaired *t*-test).
