# Supplementary material for: LPCAT1 reprogramming cholesterol metabolism promotes the progression of esophageal squamous cell carcinoma
Source: Cell Death Dis. 2021 Sep 13;12(9):845. doi: 10.1038/s41419-021-04132-6 (PMC8438019; doi:10.1038/s41419-021-04132-6)
Supplement: Supplementary file 5 — Supplemental Figure 5 [file 41419_2021_4132_MOESM5_ESM.docx]

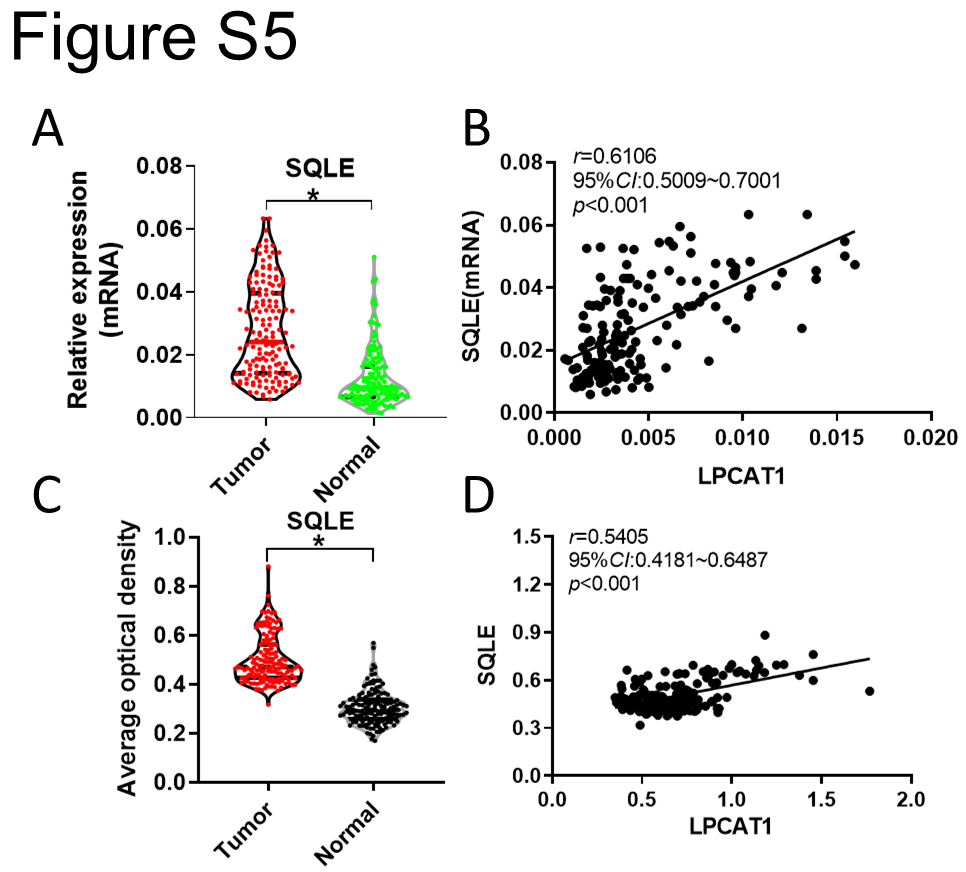


**Supplementary Figure 5.** **LPCAT1 has positive relationship with SQLE.**

**A-B**. The mRNA expression level of SQLE was detected in tumor and normal tissues by qPCR(A) and has a positive relationship with LPCAT1(B). **C-D**. The protein expression level of SQLE was detected in tumor and normal tissues by immunohistochemistry (C) and has a positive relationship with LPCAT1(D). Data are from three independent experiments. *P < 0.05, (Unpaired *t*-test, Person test).
