# Supplementary material for: LPCAT1 reprogramming cholesterol metabolism promotes the progression of esophageal squamous cell carcinoma
Source: Cell Death Dis. 2021 Sep 13;12(9):845. doi: 10.1038/s41419-021-04132-6 (PMC8438019; doi:10.1038/s41419-021-04132-6)
Supplement: Supplementary file 6 — Supplemental Figure 6 [file 41419_2021_4132_MOESM6_ESM.docx]

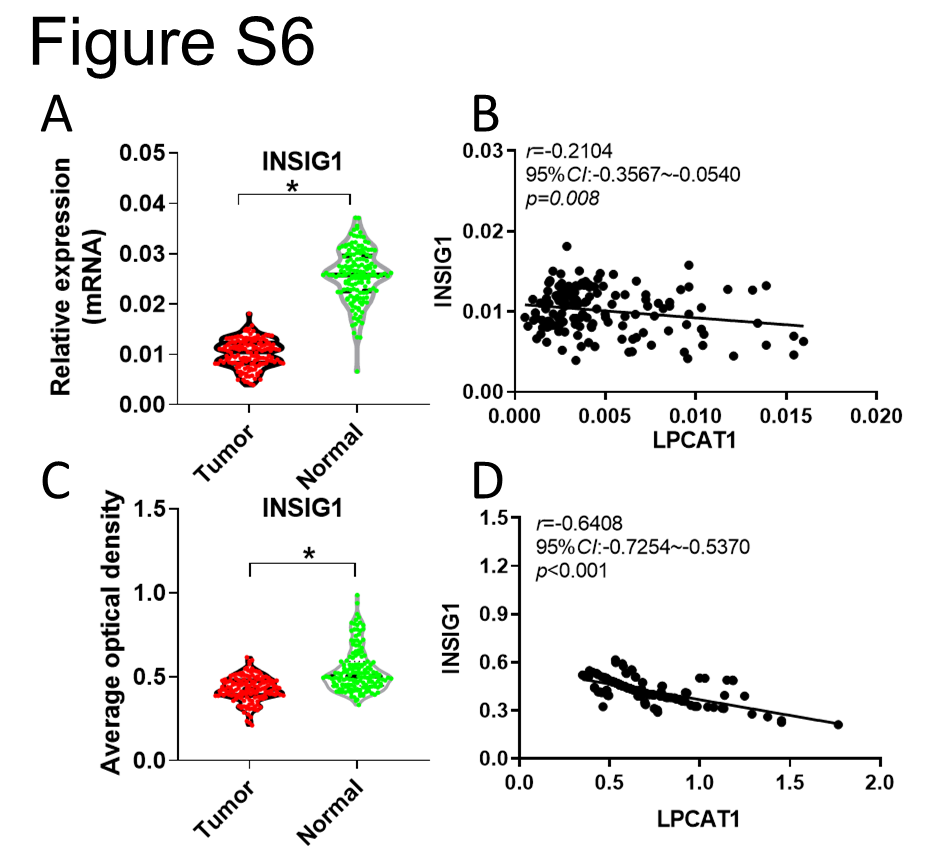


**Supplementary Figure 6. LPCAT1 has positive correlations with Insig-1 in ESCC patients.**

**A-B**. The mRNA expression level of INSIG1 was detected in tumor and normal tissues by qPCR(A) and has a positive relationship with LPCAT1(B). **C-D**. The protein expression level of INSIG1 was detected in tumor and normal tissues by immunohistochemistry (C) and has a negative relationship with LPCAT1(D). Data are from three independent experiments. *P < 0.05, (Unpaired *t*-test, Person test).
