# Supplementary material for: LPCAT1 reprogramming cholesterol metabolism promotes the progression of esophageal squamous cell carcinoma
Source: Cell Death Dis. 2021 Sep 13;12(9):845. doi: 10.1038/s41419-021-04132-6 (PMC8438019; doi:10.1038/s41419-021-04132-6)
Supplement: Supplementary file 8 — Supplemental Figure 8 [file 41419_2021_4132_MOESM8_ESM.doc]

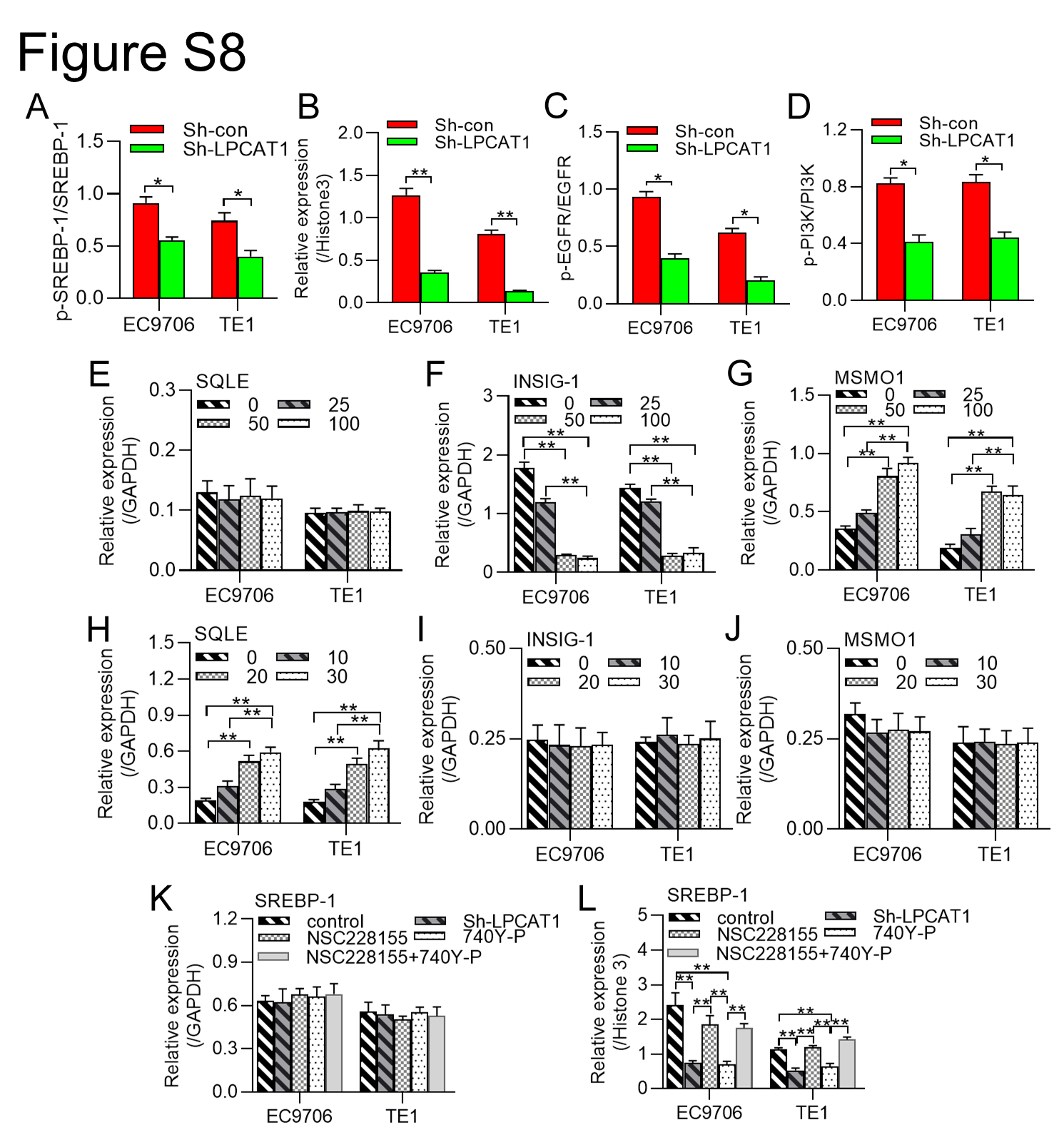


**Supplementary Figure 8. LPCAT1 promote ESCC Cholesterol synthesis by**

**EGFR/INSIG-1/SREBP-1 pathway.**

**A.** Statistical analysis of the expression of p-SREBP-1 in EC9706 and TE1 cells transfected with sh-control and sh-LPCAT1 was detected using western blot. B. Statistical analysis of the expression of SREBP-1 in nucleus in EC9706 and TE1 cells transfected with sh-control and sh-LPCAT1 was detected using western blot. **C-D.** Statistical analysis of the expression of p-EGFR and p-PI3K in EC9706 and TE1 cells transfected with sh-control and sh-LPCAT1 was detected using western blot. **E-J.** Statistical analysis of the expression of SQLE, Insig-1 and MSMO1 in EC9706 and TE1 cells treated with NSC228155 or 740Y-P after transfected with sh-control and sh-LPCAT1 was detected using western blot. **K-L.** Statistical analysis of the total and nucleus expression of SREBP-1 in EC9706 and TE1 cells treated with NSC228155 or 740Y-P after transfected with sh-control and sh-LPCAT1 was detected using western blot. Data represent three independent experiments. *P < 0.05, **P < 0.01, (Unpaired t-test, one-way ANOVA).
