# Supplementary material for: LPCAT1 reprogramming cholesterol metabolism promotes the progression of esophageal squamous cell carcinoma
Source: Cell Death Dis. 2021 Sep 13;12(9):845. doi: 10.1038/s41419-021-04132-6 (PMC8438019; doi:10.1038/s41419-021-04132-6)
Supplement: Supplementary file 9 — Supplemental Figure 9 [file 41419_2021_4132_MOESM9_ESM.docx]

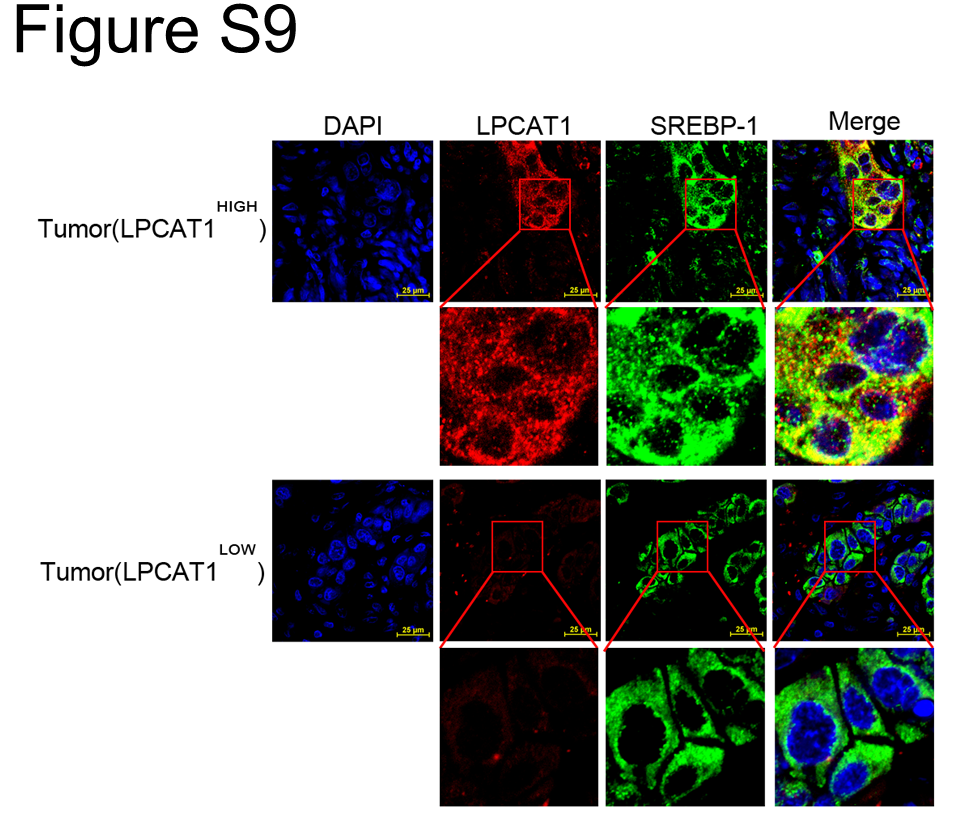


**Supplementary Figure 9. LPCAT1 co-localized with SREBP-1 in ESCC patients.**

The co-localization of SREBP1 with LPCAT1 in tumor tissues was detected by immunohistochemistry analysis. Data are from three independent experiments.
