# Supplementary material for: LPCAT1 reprogramming cholesterol metabolism promotes the progression of esophageal squamous cell carcinoma
Source: Cell Death Dis. 2021 Sep 13;12(9):845. doi: 10.1038/s41419-021-04132-6 (PMC8438019; doi:10.1038/s41419-021-04132-6)
Supplement: Supplementary file 10 — Supplemental Figure 10 [file 41419_2021_4132_MOESM10_ESM.doc]

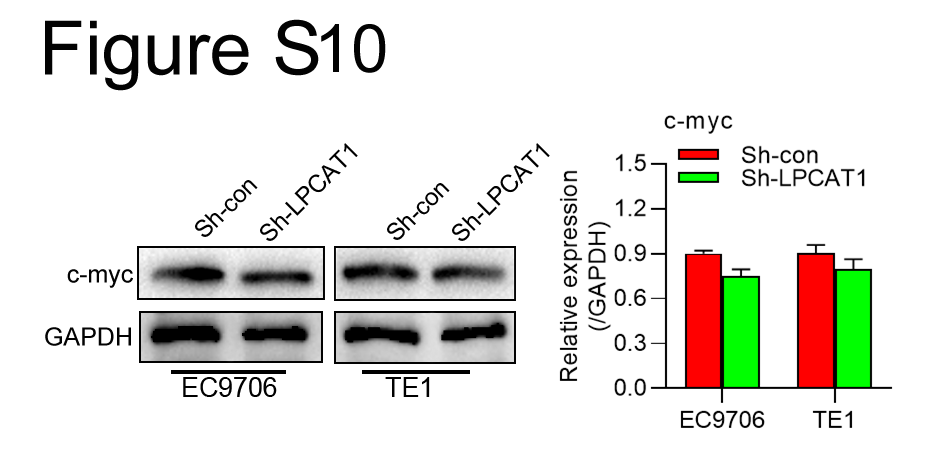


**Supplementary Figure 10. c-myc was not regulated by LPCAT1 in ESCC cells.**

TE1 cells transfected with sh-control and Sh-LPCAT1 vectors detected by western blot. Data are from three independent experiments and presented as mean ± SD
