# Supplementary material for: LPCAT1 reprogramming cholesterol metabolism promotes the progression of esophageal squamous cell carcinoma
Source: Cell Death Dis. 2021 Sep 13;12(9):845. doi: 10.1038/s41419-021-04132-6 (PMC8438019; doi:10.1038/s41419-021-04132-6)
Supplement: Supplementary file 11 — Supplemental Figure 11 [file 41419_2021_4132_MOESM11_ESM.doc]

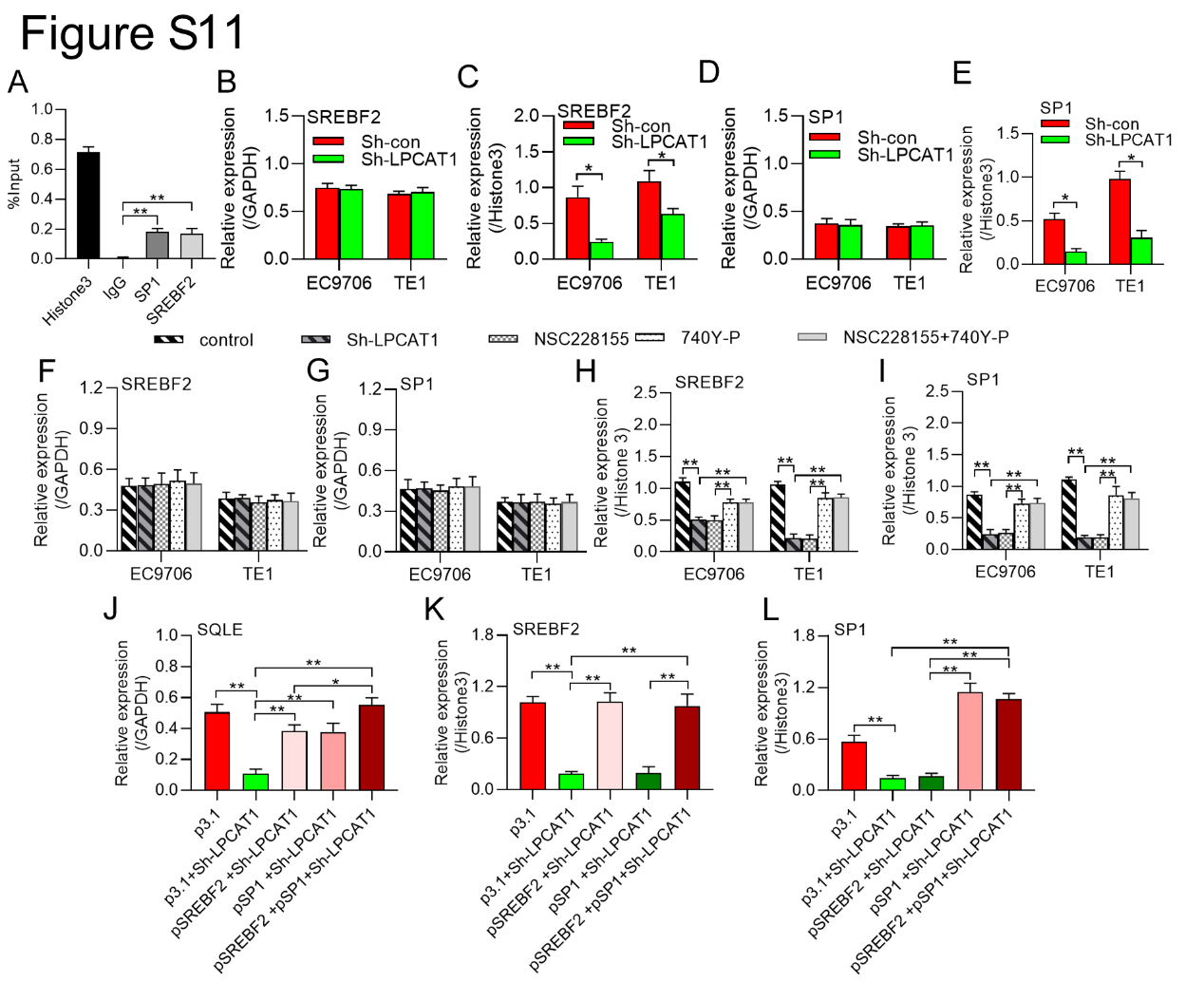


**Supplementary Figure 11. PI3K signaling promotes Cholesterol synthesis by upregulation SQLE transcriptional activity via SP1 and SREBF2.**

**A.** RT-PCR detect of SP1 and SREBF2 binding in the promoter of SQLE. **B-E.** Statistical analysis of the total and nucleus expression of SP1 and SREBF2 in EC9706 and TE1 cells was detected using western blot after knocking down of LPCAT1. **F-I.** Statistical analysis of the total and nucleus expression of SREBF2 and SP1 in EC9706 and TE1 cells treated with NSC228155 or 740Y-P after transfected with sh-control and sh-LPCAT1 was detected using western blot. **J-L.** Statistical analysis of the expression of SQLE and nucleus SP1 and SREBF2 after overexpression of SP1 and SREBF2 in TE1 cells that transfected with sh-control and sh-LPCAT1 was detected using western blot. Data are from three independent experiments. *P < 0.05, **P < 0.01. (Unpaired t-test, one-way ANOVA).
