# Supplementary material for: LPCAT1 reprogramming cholesterol metabolism promotes the progression of esophageal squamous cell carcinoma
Source: Cell Death Dis. 2021 Sep 13;12(9):845. doi: 10.1038/s41419-021-04132-6 (PMC8438019; doi:10.1038/s41419-021-04132-6)
Supplement: Supplementary file 12 — Supplemental Figure 12 [file 41419_2021_4132_MOESM12_ESM.doc]

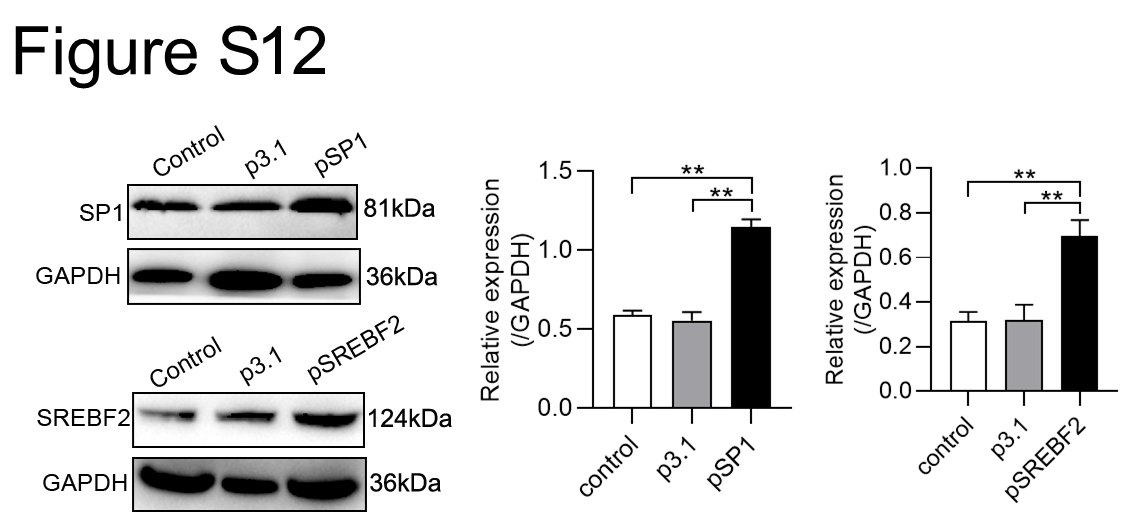


**Supplementary Figure 12. LPCAT1 co-localized with SREBP-1 in ESCC patients.**

TE1 cells transfected with pCDNA3.1, pSP1 and pSREBF2 vectors detected by western blot. Data are from three independent experiments. *P < 0.05, **P < 0.01. (one-way ANOVA).
