# Supplementary material for: LPCAT1 reprogramming cholesterol metabolism promotes the progression of esophageal squamous cell carcinoma
Source: Cell Death Dis. 2021 Sep 13;12(9):845. doi: 10.1038/s41419-021-04132-6 (PMC8438019; doi:10.1038/s41419-021-04132-6)
Supplement: Supplementary file 13 — Supplemental tables [file 41419_2021_4132_MOESM13_ESM.docx]

**Table S1 Primers of qRT-PCR used in this study**

|  | Forward Primer(5’-3’) | Reverse Primer(5’-3’) |
| --- | --- | --- |
| H-*GAPDH* | TCATTGACCTCAACTACATGGTTT | GAAGATGGTGATGGGATTTC |
| H-*LPCAT1* | ACCTATTCCGAGCCATTGACC | CCTAATCCAGCTTCTTGCGAAC |
| H-*INSIG1* | TACGCTGATCACGCAGTTTCT | TCAACTCCTCACAGAAGGGTA |
| H-*SQLE* | TTTCATTGGCTTCTTCTGGGC | ATATTGGTTCCTTTTCTGCGCCTC |
| H-*MSMO1* | TCGCGGCCGTTCAGAATTA | CATATTCCACAGCCAAGGATGC |

**Table S2 siRAN and shRNA used in this study**

|  | Sense（5’-3’） | Antisense（5’-3’） |
| --- | --- | --- |
| si-NC | UUCUCCGAACGUGUCACGUTT | ACGUGACACGUUCGGAGAATT |
| si-LPCAT1-1 | GAUCCAGUAUAUACGGCCUTT | AGGCCGUAUAUACUGGAUCTT |
| si-LPCAT1-2 | CCUGCCUAAUUACCUUCAATT | UUGAAGGUAAUUAGGCAGGTT |
| sh-NC | TTCTCCGAACGTGTCACGT |  |
| sh-LPCAT1 | CCTGCCTAATTACCTTCAA |  |

**Table S3 Primers of construction of truncated plasmids and ChIP primers used in the ChIP assay**

| Primer | Sequence (5’-3’) |
| --- | --- |
| H-*SQLE*-FL-F | GGggtaccATCTGGGTCTCCTAACTCTA |
| H-*SQLE*-FL-R | CCCaagctt ACTTTTATGATGATACCCTTTG |
| H-*SQLE*-F1-F | GGggtaccACACGACTATTTGACTTCT |
| H-*SQLE*-F1-R | CCCaagctt ACTTTTATGATGATACCCTTTG |
| H-*SQLE*-F2-F | GGggtaccGAATGGAAACGTTCCGACCCG |
| H-*SQLE*-F2-R | CCCaagctt ACTTTTATGATGATACCCTTTG |
| H-*SQLE*-F3-F | GGggtaccGCCATCTCGGCCTACCGCGC |
| H-*SQLE*-F3-R | CCCaagctt ACTTTTATGATGATACCCTTTG |
| H-*SQLE*-F4-F | GGggtaccTCCTCATCTATCCAGTGAAGC |
| H-*SQLE*-F4-R | CCCaagctt ACTTTTATGATGATACCCTTTG |
| H-*SQLE*-F3A-F | GGggtacc TAGGGGGCGGGGCAGCAC |
| H-*SQLE*-F3A-R | CCCaagcttCCAACCTAAAGATTTAGAGGC |
| H-*SQLE*-F3B-F | GGggtaccAACTTTCGTGTCTTTTTG |
| H-*SQLE*-F3B-R | CCCaagcttCCAACCTAAAGATTTAGAGGC |
| H-*SQLE*- del(F3A-F3B)-F | **GCGGCGGCAGGAGCAGGG**AACTTTCGTGTCTTTTTG |
| H-*SQLE*- del(F3A-F3B)-R | CCGCCGGCCAATGAGGGCG |
| ChIP- *SQLE* promoter-F | CGGCAGGAGCAGGGGTAGG |
| ChIP- *SQLE* promoter-R | CATGAAGCTATGTAAACGAGCCCAC |
| H-*SP1*-F | CTAgctagcTAGCCATGAGCGACCAAGATC |
| H-*SP1*-R | GCtctagaGCCTTGGACCCATGCTACCT |
| H-*SREBF2*-F | CTAgctagcTAGGGGCGATGGACGACAGCG |
| H- *SREBF2*-R | GCtctagaGCTCAGGAGGCGGCAATGGCA |

**The uppercase letters in bold were representative of homogenous arms during homologous reorganization**

**Table S4 Primers of transcription factor binding site deletion**

| **primers** | **Sequence (5’-3’)** |
| --- | --- |
| mut-SP9 -F | **TCCCTCGTCTGGGCCGAG**ATGATCCAACTT |
| mut-SP9-R | TCAGTTGGCTCAGGCGCGA |
| mut-SREBF2-F | **CGGGGCGCGCGGTATG**ATGATACTCGCGCCT |
| mut-SREBF2-R | ACTATGGGCTCGGCCCAGA |
| mut-SP1-F | **TCGCGCCTGAGCC**AACTGACCGGTCCCTCGTCT |
| mut-SP1-R | TACGGGGTCGCCTAGGAGG |
| mut-E2F1-F | **GGGGCAGCACGCGGG**ATCATCGGTATGGGC |
| mut- E2F1-R | GCTATCGTGGGGATGGGCGC |
| mut-KLF15-F | **CAGGAGCAGGGGTAGGG**ACTACGGCAGCACG |
| mut- KLF15-R | ACTATGGGCTCGGCCCAGA |

**The mutated base was underlined in the primer, and the uppercase letters in bold were representative of homogenous arms during homologous reorganization**
